# Glucosinolate-Derived Metabolites from *Barbarea vulgaris* (Brassicaceae): Evaluation of Antimicrobial, Antioxidant, and Anti-Inflammatory Potentials

**DOI:** 10.3390/molecules30234606

**Published:** 2025-11-30

**Authors:** Elvira Mavrić-Scholze, Amina Gusinac, Milan Dekić, Ivan Palić, Edina Avdović, Dušica Simijonović, Mirjana Grujović, Katarina Marković, Vladimir Dobričić, Jelena Bošković, Zoran Marković, Niko Radulović

**Affiliations:** 1 Department for Applied Biosciences and Process Engineering, Anhalt University of Applied Sciences, Bernburger Str. 55, 06366 Köthen, Germany; elvira.mavric-scholze@hs-anhalt.de; 2 Department of Chemistry, Faculty of Sciences and Mathematics, University of Niš, Višegradska 33, 18106 Niš, Serbia; aminag5667@gmail.com (A.G.); ivanpalic@yahoo.com (I.P.); nikoradulovic@yahoo.com (N.R.); 3 Department of Sciences and Mathematics, State University of Novi Pazar, Vuka Karadžića 9, 36300 Novi Pazar, Serbia; zmarkovic@uni.kg.ac.rs; 4 Institute for Information Technologies, University of Kragujevac, Jovana Cvijića bb, 34000 Kragujevac, Serbia; edina.avdovic@pmf.kg.ac.rs (E.A.); dusica.simijonovic@uni.kg.ac.rs (D.S.); mirjana.grujovic@pmf.kg.ac.rs (M.G.); katarina.mladenovic@pmf.kg.ac.rs (K.M.); 5 Department of Pharmaceutical Chemistry, University of Belgrade-Faculty of Pharmacy, Vojvode Stepe 450, 11221 Belgrade, Serbia; vladimir.dobricic@pharmacy.bg.ac.rs (V.D.); jelena.boskovic@pharmacy.bg.ac.rs (J.B.)

**Keywords:** *Barbarea vulgaris*, glucosinolate degradation products, antimicrobial activity, antioxidant activity, anti-inflammatory activity

## Abstract

Glucosinolate-derived metabolites play central roles in plant defense and are increasingly recognized for their pharmacological importance. *Barbarea vulgaris* produces a structurally diverse set of such compounds, yet their biological activities remain insufficiently explored. In this study, natural metabolites and their synthetic analogues were evaluated for antimicrobial, antibiofilm, antioxidant, and anti-inflammatory properties. Antimicrobial activity was assessed against human and plant pathogens by determining minimum inhibitory and minimum microbicidal concentrations, antibiofilm potential was examined using microplate assays, and radical scavenging activity was measured by DPPH and ABTS assays. In addition, the compounds were screened for inhibitory effects on lipoxygenase (LOX) and cyclooxygenase-2 (COX-2). Phenolic derivatives, particularly methyl-4-hydroxyphenylethyl dithiocarbamate (**2**) and 2-(4-hydroxyphenyl)ethyl isothiocyanate (**8**), exhibited notable in vitro antibacterial activity (MIC 0.312–1.25 mg mL^−1^ against *E. coli* ATCC 25922 and *S. aureus* ATCC 25923) and detectable antibiofilm effects. Racemic barbarin (**4**) preferentially inhibited LOX, underscoring its potential as an anti-inflammatory scaffold, whereas COX-2 inhibition was weak across all tested compounds. None of the metabolites showed radical scavenging activity, suggesting that their effects rely on enzyme inhibition or microbial interactions rather than nonspecific antioxidant mechanisms. This study provides an integrated evaluation of *B. vulgaris* metabolites, highlighting their ecological role in plant defense and their potential as scaffolds for novel antimicrobial and anti-inflammatory agents.

## 1. Introduction

The mustard family (Brassicaceae) is renowned for its remarkable chemical diversity, with glucosinolates (GSLs) and their hydrolysis products as defining features. Upon tissue disruption, GSLs are enzymatically cleaved by myrosinases to yield isothiocyanates, nitriles, thiocyanates, and related heterocyclic derivatives. These compounds are pivotal in plant defense, acting as feeding deterrents and antimicrobial agents [1]. In humans, cruciferous vegetables are associated with protective effects against cardiometabolic, neurological, and musculoskeletal disorders, as well as various cancers, largely due to bioactive metabolites formed through GSL hydrolysis [2]. Consequently, GSL-derived metabolites have been intensively studied for their ecological and pharmacological significance.

*Barbarea vulgaris* W.T. Aiton (winter cress) has emerged as a key model species for exploring chemical diversity in crucifers and its ecological implications. Natural populations display pronounced chemical variation, producing distinct chemotypes that differ in glucosinolate composition and ecological interactions. Besides glucosinolates, *B. vulgaris* synthesizes a broad spectrum of secondary metabolites, including saponins, flavonoid glycosides, and unique phytoalexins such as nasturlexins [3,4,5,6,7]. Collectively, these compounds form a versatile defensive arsenal that enhances the resilience of this crucifer against biotic and abiotic stress. They are increasingly recognized not only as ecological mediators but also as valuable scaffolds for drug discovery and crop protection.

Phytoalexins, low-molecular-weight secondary metabolites synthesized in response to stress, are noteworthy for their role in pathogen resistance and pronounced antimicrobial properties, particularly antifungal effects [8]. In crucifers, *B. vulgaris* produces nasturlexins alongside indole-derived compounds such as brassinin, cyclobrassinin, and cyclonasturlexin. While their chemical diversity and biosynthetic pathways are relatively well established, the biological significance of these metabolites—especially the non-indolyl nasturlexins—remains poorly understood [7,9,10,11,12,13]. Their inducible biosynthesis underscores the adaptive capacity of *B. vulgaris* and the complexity of its defense system.

In our previous work, we profiled volatile glucosinolate hydrolysis products in a Serbian population of *B. vulgaris* [14]. We observed organ-specific metabolic partitioning, discordance between chemotype and morphotype, and storage-driven isomerization of (*S*)-barbarin to its thiazolidinone analogue. Several key metabolites—including isothiocyanates, oxazolidinones, thiazolidinones, and nasturlexins—were isolated or synthesized as natural counterparts, providing a detailed chemical framework for GSL turnover. Nevertheless, knowledge of their biological activities lags behind their chemical characterization, leaving an important gap.

One promising research direction is the anti-inflammatory potential of these metabolites. Cyclooxygenase-2 (COX-2) and lipoxygenase (LOX), central enzymes of the arachidonic acid cascade, are validated therapeutic targets, and several GSL-derived isothiocyanates (e.g., sulforaphane, phenylethyl isothiocyanate) have been reported as natural inhibitors [2,15]. Similarly, antioxidant activity is often considered a hallmark of plant metabolites. Although isothiocyanates are not classical antioxidants, hydroxylated aromatic derivatives may contribute radical scavenging potential. Finally, antimicrobial and antibiofilm properties of GSL-derived compounds are ecologically relevant in plants and attractive as potential strategies against antibiotic resistance in humans [16,17].

Despite extensive chemical profiling, the biological significance of *B. vulgaris* metabolites remains poorly defined. To address this gap, the present study provides the first integrated evaluation of the biological activities of selected natural metabolites and their synthetic analogues. We investigated their antimicrobial and antibiofilm activity against human and plant pathogens, antioxidant potential using DPPH and ABTS assays, and anti-inflammatory properties via LOX and COX-2 inhibition. This combined approach bridges ecological and pharmacological perspectives, offering a comprehensive account of the bioactivity of *B. vulgaris* metabolites.

## 2. Results and Discussion

### 2.1. Isolation of Glucosinolate-Derived Metabolites from B. vulgaris and Synthesis of Equivalents and Analogues

Several metabolites previously identified in *B. vulgaris* autolysates—including isothiocyanate, oxazolidinone, thiazolidinone, oxazolidinthione, and phytoalexins—were obtained either by direct isolation from plant material or through synthesis of their natural counterparts and structural analogues to secure sufficient amounts for biological evaluation. The synthetic approach proved particularly important for metabolites occurring in low natural abundance or those prone to instability under conventional isolation conditions.

Comprehensive structural characterization of all isolated and synthesized compounds was performed using IR, MS, and NMR spectroscopy. Detailed NMR analyses—including 1D (^1^H, ^13^C, and selective homonuclear decoupling experiments) and 2D techniques (COSY, HSQC, HMBC, and NOESY), complemented by DEPT-45, DEPT-90, and DEPT-135 experiments—enabled unambiguous identification and complete assignment of proton and carbon resonances. Diagnostic IR absorptions and characteristic MS fragment ions further corroborated the proposed structures and confirmed the identity of the compounds.

**Figure 1:**
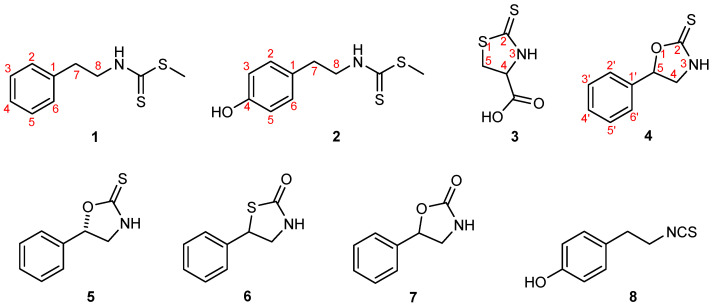
Structures of the glucosinolate-derived metabolites isolated from *B. vulgaris*, along with their synthetic equivalents and analogues investigated in this study: nasturlexin A (**1**), methyl-4-hydroxyphenylethyl dithiocarbamate (**2**), raphanusamic acid (**3**), (±)-barbarin (**4**), (*S*)-barbarin (**5**), 5-phenyl-1,3-thiazolidin-2-one (**6**), resedine (**7**), and 2-(4-hydroxyphenyl)ethyl isothiocyanate (**8**). Compounds **1**, **3**, **4** (examined as a racemic mixture of naturally occurring epimers) and **5**–**8** represent natural metabolites of *B. vulgaris*, obtained either as isolated natural products or as synthetic equivalents, whereas compound **2** is a synthetic analogue.

The structures of the glucosinolate-derived metabolites isolated from *B. vulgaris*, together with their synthetic equivalents and analogues examined in this study—nasturlexin A (**1**); methyl-4-hydroxyphenylethyl dithiocarbamate (**2**, a 4-hydroxy derivative of **1** and the proposed biosynthetic precursor of the phytoalexin tridentatol C from *Nasturtium officinale*); raphanusamic acid (**3**); (±)-barbarin (4); (*S*)-barbarin (**5**); 5-phenyl-1,3-thiazolidin-2-one (**6**); resedine (**7**); and 2-(4-hydroxyphenyl)ethyl isothiocyanate (**8**)—are shown in Figure 1. The corresponding synthetic routes, spectral data, and further analytical details are provided in the Materials and Methods section and in the Supplementary Information.

### 2.2. Antimicrobial and Antibiofilm Activities

Antimicrobial and antibiofilm properties of glucosinolate-derived compounds are of considerable ecological and pharmacological relevance. In plants, they function as chemical defenses against pathogens, whereas in humans they may inspire novel antimicrobial strategies in the face of rising antibiotic resistance. Previous studies have demonstrated that certain isothiocyanates and phytoalexins possess potent antibacterial and antifungal activities, in some cases accompanied by the ability to disrupt biofilm formation [16,17]. Because biofilms are notoriously difficult to eradicate and contribute to persistent infections, antibiofilm activity represents a particularly valuable trait. Assessing these effects against both human pathogens and phytopathogens provides a dual perspective: the former emphasizes therapeutic potential, while the latter highlights ecological function in *B. vulgaris*.

#### 2.2.1. Antimicrobial Activity

The in vitro antibacterial and antifungal activities of the isolated and synthesized compounds were evaluated by determining the minimum inhibitory concentrations (MICs) and the corresponding minimum microbicidal concentrations (MMCs; encompassing minimum bactericidal concentrations (MBCs) or minimum fungicidal concentrations (MFCs)). Antimicrobial activities against selected human pathogens and phytopathogens are summarized in Table 1, whereas the activities of reference antibiotics and antifungals are shown in Table 2. Tested concentrations ranged from 5 mg/mL to 0.039 mg/mL. The intensity of antimicrobial activity varied depending on both the compound and the microorganism species.

Among the tested compounds, **2** and **8** exhibited the most pronounced antibacterial activity. The MIC values of **8** ranged from <0.039 to 0.312 mg/mL, while those for **2** ranged between 0.078 and 0.625 mg/mL. Within the group of Gram-positive bacteria, **8** showed notable efficacy against *B. animalis* subsp. *lactis* (MIC < 0.039 mg/mL) and *S. aureus* (MIC 0.078 mg/mL). Among Gram-negative bacteria, notable activity was observed against *E. coli* (MIC 0.039 mg/mL), *E. coli* ATCC 25922 (MIC 0.078 mg/mL) and *P. mirabilis* (MIC 0.156 mg/mL). Compound **2** also demonstrated broad-spectrum antimicrobial activity, effectively inhibiting Gram-negative strains such as *P. mirabilis* (MIC 0.156 mg/mL), *E. coli*, and *E. coli* ATCC 25922 (both MIC and MBC 0.312 mg/mL). Even greater efficacy was observed against Gram-positive strains, particularly *B. subtilis* ATCC 6633 and *B. animalis* subsp. *lactis* (MIC 0.078 mg/mL).

Compound **3** exhibited significant antibacterial activity, with MIC values ranging from 0.156 to 2.5 mg/mL. The lowest MIC was recorded against *S. aureus* (0.156 mg/mL), while *S. aureus* ATCC 25923, *B. animalis* subsp. *lactis*, *B. pumilus* NCTC 8241, and *E. coli* ATCC 25922 showed MIC values of 0.625 mg/mL. Compound **7** exhibited moderate antimicrobial activity, with MIC values ranging from 1.25 to 5 mg/mL. It was most effective against Gram-negative bacteria (*E. coli*, *E. coli* ATCC 25922, and *S. enterica*, MIC 1.25 mg/mL), and moderately active against Gram-positive strains such as *S. aureus* and *B. animalis* subsp. *lactis* (MIC 2.5 mg/mL).

The racemic form of barbarin (**4**) showed moderate antibacterial activity, with MIC values of 1.25–5 mg/mL, being most effective against *S. aureus*, *S. aureus* ATCC 25923, *B. animalis* subsp. *lactis*, and *B. pumilus* NCTC 8241 (MIC 1.25 mg/mL). Compound **1** also exhibited moderate activity (MIC 1.25–5 mg/mL), with *B. pumilus* NCTC 8241, *B. animalis* subsp. *lactis*, and *E. coli* ATCC 25922 among the most sensitive strains (MIC 1.25 mg/mL). The natural form of barbarin (**5**) demonstrated more selective and strain-dependent antibacterial effects. In contrast, all tested bacterial strains were resistant to compound **6**, which showed no detectable antibacterial activity at the tested concentrations.

The tested fungal strains exhibited the highest sensitivity to isothiocyanate **8** and compound **2**, with MIC values ranging from <0.039 to 2.5 mg/mL for **8** and 0.156 to 2.5 mg/mL for **2**. Among the tested fungi, *T. viride* ATCC 13233, *A. flavus* ATCC 9170, and *F. solani* ATCC 11712 were the most susceptible to **8**, with both MIC and MMC values recorded at <0.039 mg/mL. Compound **3** displayed the strongest antifungal effect against *C. albicans* ATCC 10231 (MIC 0.312 mg/mL), followed by **1**, which showed moderate activity (MIC 1.25 mg/mL). The antifungal activity of the remaining compounds was generally lower, displaying species-dependent and selective effects. Notably, all fungal strains were resistant to compound **6**.

In summary, glucosinolate-derived metabolites exhibited differential antimicrobial effects across bacterial and fungal strains, with pronounced variation between Gram-positive and Gram-negative bacteria. Isothiocyanate **8**, consistent with its broad-spectrum profile, was among the most effective compounds, likely due to the electrophilicity of the –N=C=S group and its reactivity with thiol and amino residues in microbial proteins. Compounds bearing phenolic groups (**2** and **8**) also showed notable activity, suggesting that hydroxylation of the aromatic ring enhances microbial interactions. Nasturlexin A (**1**) and heterocyclic derivatives such as barbarin (**4** and **5**) and its oxazolidinone analogue (**7**) demonstrated antimicrobial potential, although with narrower spectra of activity. Furthermore, *B. vulgaris* produces phenyl-containing phytoalexins, including nasturlexins C and D and their sulfoxide derivatives [7], which are active against crucifer pathogens and likely function as natural protectants [7,9,13]. These findings highlight that *B. vulgaris* synthesizes a structurally diverse set of metabolites that together contribute to enhanced pathogen resistance.

In the present work, we extended the investigation of *B. vulgaris* secondary metabolites by evaluating their in vitro antimicrobial activity not only against phytopathogens but also against clinically relevant human pathogens. To the best of our knowledge, this is the first report on the antimicrobial potential of *B. vulgaris* metabolites conducted in Serbia and among the few carried out in Europe, despite the plant’s wide natural distribution across the continent. The novelty of this study lies in its dual focus on plant and human pathogens, thereby underscoring the broader significance of *B. vulgaris* bioactives beyond agricultural applications.

These findings suggest clear structure–activity relationships. Compounds bearing phenolic substituents (**2** and **8**) were the most active, indicating that hydroxylation of the aromatic ring enhances microbial interactions, likely through hydrogen bonding and increased polarity. The notable and broad-spectrum activity of isothiocyanate **8** further reflects the electrophilicity of the –N=C=S group, which readily reacts with thiol and amino residues in microbial proteins.

#### 2.2.2. Inhibition of Biofilm Formation

Biofilm formation was quantified by crystal violet staining and absorbance measurement. Among the strains examined, *S. aureus* ATCC 25923, *B. subtilis* ATCC 6633, *P. mirabilis*, *S. enterica*, and *K. pneumoniae* demonstrated the highest biofilm-forming capacities, with absorbance values of 1.33, 0.77, 1.02, 1.19, and 1.61, respectively.

Among the tested compounds, **2** and **8** demonstrated the most prominent antibiofilm activity across Gram-positive and Gram-negative strains, as reflected by their low BIC_50_ and BIC_90_ values (Table 3). Compound 2 emerged as the most potent, with BIC_50_ values of 220 µg/mL against *S. aureus* ATCC 25923, 290 µg/mL against *B. subtilis* ATCC 6633, and 580 µg/mL against *K. pneumoniae*. Although BIC_90_ values were relatively high, compound **2** still retained measurable antibiofilm activity across several strains. Compound **8** also exhibited substantial antibiofilm efficacy, particularly against *K. pneumoniae* (BIC_50_ 570 µg/mL; BIC_90_ 2000 µg/mL) and *S. aureus* ATCC 25923 (BIC_50_ 868 µg/mL), but was less effective against *S. enterica* (no BIC_90_ reached below 5000 µg/mL), suggesting a more selective strain-specific profile compared to compound **2**.

The racemic form of barbarin (**4**) showed moderate activity, particularly against *S. enterica* (BIC_50_ 1000 µg/mL), but lacked efficacy at higher inhibitory thresholds, as most BIC_90_ values exceeded 5000 µg/mL. Similarly, compound **3** demonstrated some inhibitory effects, particularly against *B. subtilis* ATCC 6633 and *K. pneumoniae* (BIC_50_ 1190 and 1262 µg/mL), though BIC_90_ values were again elevated. In contrast, compound **7** showed the weakest activity overall, with BIC_50_ values > 5000 µg/mL for most strains and only moderate inhibition of *S. enterica* (BIC_50_ 1254 µg/mL) and *P. mirabilis* (BIC_50_ 3100 µg/mL). The lack of significant BIC_90_ effects further underscores its limited antibiofilm potential.

Biofilm formation is a major virulence factor in clinically and environmentally relevant bacteria, conferring resistance to antimicrobials and enabling persistence in both host and non-host environments [18]. In this study, we evaluated the antibiofilm activity of selected *B. vulgaris* secondary metabolites. These results provide novel insights into the bioactive properties of *B. vulgaris* and expand the understanding of its potential applications in controlling biofilm-associated microorganisms.

Of particular interest is the notable antibiofilm activity observed for several compounds. Since biofilms are notoriously resistant to eradication and contribute to chronic infections in both clinical and agricultural contexts, the ability of *B. vulgaris* metabolites to inhibit biofilm formation or disrupt established biofilms adds an important dimension to their biological profile. This property distinguishes them from conventional antimicrobials, which often fail to address biofilm-associated infections.

Moreover, by evaluating activity against both human pathogens and phytopathogens, this study provides complementary insights: activity against human-associated microbes highlights translational potential, whereas effects on phytopathogens underscore possible ecological roles in the plant’s defense strategy.

The antibiofilm results further reinforce these SAR insights: phenolic derivatives (**2** and **8**) were consistently the most effective, whereas heterocyclic scaffolds (**4**, **7**) showed weaker and more selective activity. These findings highlight phenolic substitution as a key determinant of biofilm inhibition.

### 2.3. Antioxidant Activity Against DPPH and ABTS Radicals

Antioxidant activity represents another hallmark property of many plants’ secondary metabolites. Oxidative stress is a central contributor to the pathogenesis of numerous chronic diseases, and radical scavenging capacity is frequently considered a first-line indicator of therapeutic potential. Accordingly, radical scavenging is commonly used as a preliminary screen for bioactivity in plant-derived metabolites. Although isothiocyanates are not classical antioxidants, phenolic isothiocyanates and structurally related derivatives may exert radical-scavenging effects through their hydroxylated aromatic moieties. Evaluation of antioxidant activity using widely applied assays such as DPPH and ABTS therefore provides valuable comparative insights into the functional diversity of GSL-derived compounds.

In this study, the antioxidant properties of isolated glucosinolate-derived metabolites from *B. vulgaris* and their synthetic equivalents or analogues were evaluated using two standard assays, DPPH and ABTS, which together provide a reliable measure of free-radical scavenging capacity. As presented in Table 4 and Table 5, assays were performed at concentrations of 25, 50, and 100 µM, with incubation times of 20 and 60 min for the DPPH test. None of the tested compounds showed significant DPPH or ABTS radical scavenging activity, even at the highest concentrations. These findings exclude nonspecific antioxidant mechanisms and suggest that biological effects arise from microbial interactions or enzyme inhibition.

### 2.4. In Vitro LOX and COX-2 Inhibition Assay

One promising avenue for functional studies lies in the anti-inflammatory potential of these compounds. Cyclooxygenase-2 (COX-2) and lipoxygenase (LOX) are key enzymes of the arachidonic acid cascade responsible for the generation of prostaglandins and leukotrienes, respectively. Both represent central mediators of inflammation and validated therapeutic targets. Several GSL-derived isothiocyanates, such as sulforaphane and phenylethyl isothiocyanate, have been reported to exert anti-inflammatory effects, thereby underscoring the relevance of GSL-derived metabolites as natural anti-inflammatory agents [2,15]. Nevertheless, the inhibitory potential of *B. vulgaris*-specific metabolites remains poorly understood, highlighting the gap between chemical characterization and functional validation. Here, we evaluate the inhibitory activities of these metabolites against COX-2 and LOX to gain insight into their potential anti-inflammatory roles. Both enzymes are implicated not only in inflammatory disorders but also in cardiovascular disease and cancer. Although synthetic inhibitors of COX-2 and LOX are well established in pharmacotherapy, inhibitors derived from natural products remain highly appealing owing to their structural diversity and generally favorable safety profiles.

Soybean LOX catalyzes the oxygenation of polyunsaturated fatty acids, leading to the production of lipid hydroperoxides and playing a crucial role in the biosynthesis of bioactive mediators involved in inflammation and oxidative stress. Therefore, the assessment of LOX inhibition directly indicates the anti-inflammatory potential of tested compounds. In this study, all compounds were assessed at 100 µM, and IC_50_ values were determined for the most active inhibitors. Compound **4** (racemic barbarin) exhibited the strongest activity (IC_50_ = 61.6 µM), followed by compound **2** (IC_50_ = 97.4 µM), while quercetin, used as a reference, showed the highest potency (IC_50_ = 43.2 µM). The remaining compounds were largely inactive (Table 6).

The results of DPPH and ABTS assays indicate that these compounds do not possess significant antioxidant capacity, excluding the possibility of anti-inflammatory activity via a radical scavenging mechanism [19]. Instead, they most likely act as direct enzyme inhibitors by binding at or near the active site, thereby preventing natural substrate access such as linoleic acid [19]. Further research is warranted to clarify their exact mechanism of action and to explore structure–activity relationships for the design of more potent inhibitors.

Cyclooxygenase (COX) is an enzyme essential for the production of prostanoids from arachidonic acid. Of its two isoforms, COX-1 is constitutively expressed in many tissues, whereas COX-2 is induced primarily during inflammatory responses. Based on LOX results, *rac*-barbarin (**4**) and enantiopure (*S*)-barbarin (**5**) were selected for COX-2 testing. Both compounds showed only weak COX-2 inhibition—none of the tested compounds achieved 50% COX-2 inhibition at tested concentrations—indicating low potency (IC_50_ > 100 µM) in comparison with their LOX activity.

The stronger LOX inhibition by *rac*-barbarin (**4**) compared to its enantiopure form (**5**) suggests that stereochemistry contributes to modulating activity. Racemic barbarin (**4**) displayed LOX-selective inhibition with an IC_50_ of 61.6 µM, which is only moderately weaker than the reference inhibitor quercetin (IC_50_ = 43.2 µM), yet still demonstrates a clear preference for LOX over COX-2 (IC_50_ > 100 µM). In contrast, COX-2 inhibition was uniformly weak across all tested compounds, further underscoring their selectivity for LOX. The absence of meaningful antioxidant effects supports the conclusion that their biological activities are mediated primarily through targeted enzyme inhibition rather than nonspecific radical scavenging.

Taken together, these observations underscore that *B. vulgaris* produces structurally diverse metabolites with complementary biological functions: phenolic isothiocyanates act as broad-spectrum antimicrobials and antibiofilm agents, while heterocyclic derivatives serve as selective enzyme inhibitors. This dual functionality reflects their ecological role in plant defense and highlights their translational potential as scaffolds for the development of antimicrobial and anti-inflammatory agents.

## 3. Materials and Methods

### 3.1. General Experimental Procedures

All solvents and reagents were obtained from commercial suppliers (Sigma-Aldrich, St. Louis, MO, USA; Merck, Darmstadt, Germany; or Acros Organics, Geel, Belgium) and used without further purification unless otherwise specified. Solvents dried prior to use are indicated in the corresponding experimental sections. Two hydrocarbon mixtures (Sigma-Aldrich, St. Louis, MO, USA), ranging from heptane to icosane and from heneicosane to tetracontane, were used for the determination of GC retention indices.

Melting points were determined on a Büchi Melting Point B-540 apparatus (Büchi Labortechnik AG, Flawil, Switzerland) and are reported uncorrected.

ATR-IR spectra were acquired on a Thermo Nicolet 6700 FT-IR spectrometer (Thermo Fisher Scientific, Waltham, MA, USA) using the attenuated total reflectance (ATR) mode.

All UV-Vis measurements were performed using a PerkinElmer Lambda 365 UV/Vis spectrophotometer (Waltham, MA, USA).

The determination of fluorescence was carried out using a Synergy LX multi-mode microplate reader (BioTek, Winooski, VT, USA).

The optical density (OD) of the stained biofilm was measured at 630 nm using an enzyme-linked immunosorbent assay (ELISA) plate reader (RT-2100C, Rayto, Shenzhen, China).

NMR spectra were recorded at 20 °C on a Bruker Avance III 400 MHz NMR spectrometer (Bruker, Fällanden, Switzerland) operating at 400 MHz for ^1^H and 100.6 MHz for ^13^C, equipped with a 5 mm dual ^13^C/^1^H probe. Spectra were acquired in CDCl_3_ or DMSO-d_6_ (Sigma-Aldrich, St. Louis, MO, USA), with tetramethylsilane (TMS) as the internal standard. Chemical shifts (δ) are given in ppm relative to TMS (δ_H_ 0.00), or in heteronuclear 2D spectra, relative to residual solvent signals (CDCl_3_: δ_H_ 7.26, δ_C_ 77.16; DMSO-d_6_: δ_H_ 2.51, δ_C_ 39.97). Coupling constants (J) are reported in Hertz (Hz). Signal multiplicities are abbreviated as follows: s = singlet, brs = broad singlet, d = doublet, dd = doublet of doublets, ddd = doublet of doublets of doublets, t = triplet, and m = multiplet. 2D experiments (NOESY, gradient ^1^H–^1^H COSY, HSQC, and HMBC), DEPT-45, DEPT-90, DEPT-135, and selective homonuclear decoupling experiments were conducted using standard Bruker pulse sequences.

Analytical thin-layer chromatography (TLC) was performed on precoated silica gel 60 F_254_ plates (0.20 mm, Macherey-Nagel, Düren, Germany). Spots were visualized under UV light (254 nm) and by spraying with 50% (*v*/*v*) aqueous H_2_SO_4_ followed by heating.

Preparative flash chromatography (FC) and preparative dry-flash chromatography (FDC) were carried out on silica gel 60 (particle size 0.063–0.200 mm) under isocratic and/or gradient elution as specified in the synthetic procedures.

Gas chromatography–mass spectrometry (GC/MS) analyses were performed on an Agilent 7890B GC system (Agilent Technologies, Santa Clara, CA, USA) equipped with an HP-5MS capillary column (5% phenyl/95% dimethylpolysiloxane, 30 m × 0.25 mm i.d., 0.25 μm film thickness) coupled to an Agilent 240-MS ion trap detector (Agilent Technologies, Santa Clara, CA, USA). The injector and transfer line temperatures were 250 °C and 325 °C, respectively. The oven program was 40 °C (initial), ramped to 315 °C at 5 °C min^−1^, and held for 10 min. Helium was used as the carrier gas at a constant flow of 1.0 mL min^−1^. Samples (1 μL of a 1% solution in dichloromethane (DCM) or Et_2_O) were injected in split mode (40:1). MS conditions were as follows: trap temperature 100 °C, ion source 180 °C, manifold 50 °C; ionization energy 70 eV; acquisition range *m*/*z* 35–500; scan rate 2.08 Hz. Linear retention indices (RI) were calculated relative to a homologous series of n-alkanes analyzed under identical conditions [20]. The samples were analyzed in triplicate; the relative standard deviation (RSD) of RI values did not exceed 1 RI unit.

Gas chromatography with flame ionization detection (GC–FID) was performed on an Agilent 7890A GC system (Agilent Technologies, Palo Alto, CA, USA) equipped with a single injector, a flame ionization detector (FID), and an HP-5MS fused silica capillary column (5% diphenylsiloxane, 95% dimethylsiloxane; 30 m × 0.32 mm i.d., film thickness 0.25 μm). The oven program was as follows: initial temperature 70 °C, ramped to 300 °C at 15 °C/min, followed by an isothermal hold at 300 °C for 5 min. Nitrogen was used as the carrier gas at a constant flow rate of 3.0 mL/min. The injector temperature was set to 250 °C, and injections (1.0 μL of solution) were performed in splitless mode. FID parameters were: detector temperature 300 °C, H_2_ flow 30 mL/min, air flow 400 mL/min, and makeup gas flow 23.5 mL/min. Data were collected using Agilent GC ChemStation software (ver. B.04.03, Agilent Technologies, Santa Clara, CA, USA) with a digitization rate of 20 Hz. Each sample was analyzed in triplicate. Percent composition (purity confirmations) was calculated from GC-FID peak areas without response factor corrections. The relative standard deviation (RSD) of peak areas from triplicate injections was consistently below 1%.

### 3.2. Synthesis of Nasturlexin A (Methyl phenethylcarbamodithioate) (***1***)

In 6.20 mL of pyridine, phenylethylamine (0.250 mL, 2 mmol) was added at 0 °C, immediately followed by carbon disulfide (2.0 mL, 33.1 mmol) and triethylamine (0.8 mL, 5.74 mmol). The reaction mixture was stirred at room temperature for 30 min, after which methyl iodide (0.181 mL, 2.10 mmol) was added, and stirring continued for an additional 15 min. Toluene was then added to the reaction mixture, and the solution was concentrated *in vacuo*. The resulting residue (1.3 g) was purified by flash-dry chromatography (hexane/EtOAc 9:1) to afford 328 mg of nasturlexin A. The purity of the product was confirmed by TLC and GC–MS analyses (partial decomposition was observed in the GC inlet). The spectral data were consistent with previously published values [10]. The corresponding synthetic route, spectral data, and additional analytical details are provided in the Supplementary Information.

### 3.3. Synthesis of Methyl-4-hydroxyphenylethyl Dithiocarbamate (***2***)

In 3.75 mL of pyridine, 250 mg of tyramine was dissolved at 0 °C, followed by the addition of carbon disulfide (1.25 mL, 20.7 mmol) and triethylamine (0.5 mL, 3.59 mmol). The reaction mixture was stirred at room temperature for 30 min, after which methyl iodide (0.125 mL, 2.01 mmol) was added and stirring continued for an additional 15 min. The mixture was then treated with toluene and concentrated *in vacuo*. The resulting solid was purified by flash dry chromatography using hexane/EtOAc (1:1) as the eluent, affording 330 mg of methyl-4-hydroxyphenylethyl dithiocarbamate (**2**). The purity of the product was confirmed by TLC and GC–MS analyses (the product completely decomposed to the corresponding isothiocyanate in the GC inlet). The spectral data were consistent with previously published values [10,21]. The corresponding synthetic route, spectral data, and additional analytical details are provided in the Supplementary Information.

### 3.4. Synthesis of Raphanusamic Acid (2-Thioxothiazolidine-4-carboxylic Acid) (***3***)

A solution of NaOH (0.8 g) in distilled water (15 mL) was prepared, to which L-cysteine hydrochloride monohydrate (0.875 g) was added. Carbon disulfide (0.325 mL) was then introduced, and the mixture was stirred until complete dissolution. Subsequently, a mixture of FeSO_4_ (0.765 g) and NaOH (0.2 g) was added, and the reaction was stirred for 2 h. The resulting solid was filtered and washed with water. The filtrate was acidified to pH 2–3, extracted with EtOAc, dried over anhydrous Na_2_SO_4_, and concentrated *in vacuo*. To afford 263 mg of raphanusamic acid. The purity of the product was confirmed by TLC and GC–MS analyses. The spectral data were consistent with previously published values [22,23,24]. The corresponding synthetic route, spectral data, and additional analytical details are provided in the Supplementary Information.

### 3.5. Synthesis of (±)-Barbarin (rac-5-Phenyl-1,3-oxazolidine-2-thione) (***4***) [25]

In 3.3 mL of anhydrous ethanol, anhydrous potassium carbonate (230 mg), carbon disulfide (0.4 mL), and rac-1-amino-2-phenylethanol (460 mg) were combined, followed by the careful dropwise addition of hydrogen peroxide (0.566 mL). The reaction mixture was heated in a water bath at 50 °C. The initial light-yellow coloration disappeared within a few seconds, and a precipitate soon formed, which was removed by filtration. The filtrate was extracted with ethyl acetate (100 mL), and the organic layer was washed with saline (3 × 50 mL), dried over anhydrous Na_2_SO_4_, and concentrated in vacuo to give 462 mg of a dry residue. Purification by preparative flash chromatography (hexane/EtOAc, 3:1) afforded 260 mg of rac-5-phenyl-1,3-thiazolidin-2-one (**4**). The purity of the product was confirmed by TLC and GC–MS analyses. The spectral data were consistent with previously published values [14,26]. The corresponding synthetic route, spectral data, and additional analytical details are provided in the Supplementary Information.

### 3.6. Isolation of (S)-Barbarin (***5***) and Determination of Absolute Configuration

(*S*)-Barbarin ((*S*)-5-phenyl-1,3-oxazolidine-2-thione, **5**) was isolated from an autolysate sample as described in the Supplementary Materials of a manuscript currently under review [14]. The absolute configuration of **5** was determined by ^1^H NMR titration with a chiral lanthanide shift reagent. For completeness, the full experimental procedure, as well as spectral and other analytical data, are provided in the Supplementary Information of this work.

### 3.7. Isolation of 5-Phenyl-1,3-thiazolidin-2-one (***6***)

5-Phenyl-1,3-thiazolidin-2-one (**6**) was isolated from an autolysate sample as reported in the Supplementary Materials of a manuscript under review [14]. For completeness, the full procedure, spectral and other analytical data are also included in the SI of this work.

### 3.8. Synthesis of Resedine (5-Phenyl-1,3-oxazolidin-2-one, ***7***)

Synthesis of 5-phenyl-1,3-oxazolidin-2-one (resedine, **7**) was synthesized as reported in the Supplementary Materials of a manuscript under review [14]. For completeness, the full procedure, spectral and other analytical data are also included in the SI of this work.

### 3.9. Synthesis of 2-(4-Hydroxyphenyl)ethyl Isothiocyanate (***8***)

2-(4-Hydroxyphenyl)ethyl isothiocyanate (**8**) was synthesized as reported in the Supplementary Materials of a manuscript currently under review [14]. For completeness, the full procedure, spectral and other analytical data are also included in the SI of this work.

### 3.10. In Vitro Antimicrobial Assay

#### 3.10.1. Microorganisms Used for Testing

The antimicrobial activity was evaluated against 15 microorganisms. This included 11 bacterial strains: four standard strains (*Staphylococcus aureus* ATCC 25923, *Bacillus subtilis* ATCC 6633, *Bacillus pumilus* NCTC 8241, and *Escherichia coli* ATCC 25922) and six clinical or environmental isolates (*Staphylococcus aureus*, *Escherichia coli*, *Bacillus cereus*, *Proteus mirabilis*, *Salmonella enterica*, and *Klebsiella pneumoniae*). One probiotic strain, *Bifidobacterium animalis* subsp. *lactis* was tested. In addition, four fungal strains, *Candida albicans* ATCC 10231, *Trichoderma viride* ATCC 13233, *Aspergillus flavus* ATCC 9170, and *Fusarium solani* ATCC 11712 were tested. The bacterial strains were kept in glycerol stock at −80 °C and the fungal strains in paraffin oil stock at 4 °C.

#### 3.10.2. Preparation of Suspensions

Bacterial and yeast suspensions were prepared using the direct colony method, as previously described by Andrews [27]. The turbidity of the initial suspensions was adjusted to match the 0.5 McFarland standard using a densitometer (Biosan, Riga, Latvia), corresponding to approximately 10^8^ colony-forming units (CFU)/mL for bacterial cells and 10^6^ CFU/mL for yeast cells. Subsequently, 1:100 dilutions of the initial suspensions were prepared in sterile 0.85% saline. Fungal spore suspensions were obtained by gently harvesting spores from agar slopes containing mature mycelial growth. The collected spore suspensions were then diluted 1:1000 in sterile 0.85% saline prior to use.

#### 3.10.3. Microdilution Method

Antimicrobial activity was evaluated by determining the minimum inhibitory concentration (MIC) and minimum microbicidal concentration (MMC) using the microdilution plate method with resazurin as an indicator [28]. In the 96-well plates, 100 μL of Mueller–Hinton broth (Torlak, Belgrade, Serbia) for bacterial testing and Sabouraud broth (Torlak, Belgrade, Serbia) for fungal testing were dispensed into each well. The compounds under investigation were initially dissolved in a 10% dimethyl sulfoxide (DMSO) solution, followed by dilution in sterile distilled water, resulting in a working compound solution containing 1% DMSO. A 100 μL aliquot of the stock solution of the tested compound was added to the first row, and twofold serial dilutions were then carried out using a multichannel pipette. The resulting concentration range was from 5 to 0.039 mg/mL. The inoculated microtiter plates were incubated at 37 °C for 24 h for bacteria, at 28 °C for 48 h for yeasts, and at 28 °C for 72 h for molds.

Each test also included growth control and sterility control. All tests were conducted in duplicate. MIC was defined as the lowest concentration of tested compounds that prevented resazurin color change from blue to pink. For molds, MIC values of the tested compound were determined as the lowest concentration that inhibited visible mycelia growth. MMC was determined by inoculation of the nutrient agar medium by plating 10 mL of samples from wells, where no indicator color change was recorded. At the end of the incubation period, the lowest concentration with no growth (no colony) was defined as MMC.

Doxycycline, ampicillin and amphotericin B (- Sigma-Aldrich, St. Louis, MO, USA) and ketoconazole (Hemofarm A.D., Vršac, Serbia) were dissolved in a nutrient liquid medium. Doxycycline is a broad-spectrum antibiotic effective against both aerobic and anaerobic Gram-positive and Gram-negative bacteria. Ampicillin, a beta-lactam antibiotic, primarily targets Gram-positive bacteria but also some Gram-negative strains. Amphotericin B is an antifungal drug often used for serious systemic fungal infections and is one of the effective treatments for fungal infections caused by *Aspergillus* sp. Ketoconazole is an antifungal agent used to inhibit the growth of *Candida* species. The negative control (diluted form of 1% DMSO) did not show activity on microbial growth.

### 3.11. Determination of Antibiofilm Activity

#### 3.11.1. Biofilm Formation by Tested Bacteria

The biofilm-forming abilities of *S. aureus* ATCC 25923, *B. subtilis* ATCC 6633, *P. mirabilis*, *S. enterica*, and *K. pneumoniae* were assessed based on the method described by O’Toole et al., with some modifications [29].

The procedure involved preparing 96-well microtiter plates (Sarstedt, Nümbrecht, Germany) by adding 100 μL of Mueller-Hinton nutrient broth to each well. A 10 μL aliquot of bacterial suspension (adjusted to 1.0 McFarland for Gram-positive bacteria and 0.5 McFarland for Gram-negative bacteria) was added to the wells. The plates were then incubated at 37 °C for 48 h to allow biofilm formation. After incubation, the wells were washed twice with 200 μL of sterile 0.85% saline to remove non-adherent and weakly adherent cells. Biofilms formed by the adherent bacteria were fixed with 100 μL of methanol until fully evaporated. The biofilms were then stained using 0.1% (*w*/*v*) crystal violet (Fluka Chemie AG, Buchs, Switzerland) and left at room temperature for 20 min. After staining, excess dye was washed off with deionized water, followed by 96% ethanol. Controls included wells containing only nutrient broth to check for sterility and nonspecific binding. Background absorbance from the sterile medium, fixative, and dye was subtracted from the OD readings of the test samples. All experiments were performed in triplicate.

#### 3.11.2. Inhibition of Biofilm Formation

To assess the inhibition of biofilm formation, 96-well microtiter plates (Sarstedt, Nümbrecht, Germany) were prepared by dispensing 100 μL of Mueller-Hinton broth into each well. From stock solutions of tested compounds (at 10 mg/mL), 100 μL was added to the first row of the plate, followed by twofold serial dilutions (from 5 to 0.039 mg/mL) using a multichannel pipette (Eppendorf AG, Hamburg, Germany). Subsequently, 10 μL of fresh bacterial suspension (adjusted to 0.5 McFarland for Gram-negative and 1.0 McFarland for Gram-positive bacteria) was added to each well. The plates were incubated at 37 °C for 48 h. After incubation, the contents of the wells were carefully removed by tapping, and the remainder of the experiment was conducted following the procedure described above.

The biofilm inhibitory concentration (BIC_50_) was defined as the lowest concentration of tested compounds that resulted in a 50% reduction in biofilm formation, as described by Chaieb et al. and Muruzović et al. [30,31]. Controls consisted of wells containing only broth or broth with the tested compounds to assess sterility and nonspecific binding. Background absorbance was subtracted from test readings as described earlier. All experiments were performed in triplicate, and tetracycline (Sigma-Aldrich, St. Louis, MO, USA), dissolved in nutrient broth, was used as a reference compound.

### 3.12. In Vitro Tests for the Assessment of Antioxidant Activity

#### 3.12.1. DPPH Radical Scavenging Assay

The DPPH assay was used to assess the ability of the tested compounds to scavenge reactive radical species [32]. Stock solutions of the investigated compounds were prepared in DMSO and then diluted with methanol to a final volume of 1000 µL. An equal volume of a 50 µM DPPH solution in methanol was then added to the prepared mixture. The final concentrations of the test compounds were 25, 50, and 100 µM. After thorough mixing, the samples were incubated in the dark at room temperature for 20 to 60 min. Methanol was used as a blank, and absorbance was measured at 517 nm after incubation. All measurements were performed in triplicate [32].

#### 3.12.2. ABTS Radical Scavenging Assay

The ABTS radical cation solution was prepared following the previously described protocol [32]. Different concentrations of the analyzed samples were prepared in DMSO. The test samples were prepared following the specified procedure: 20 µL of sample was combined with 980 µL of methanol, followed by the addition of 1000 µL of ABTS solution. Absorbance was measured at 734 nm. All measurements were performed in triplicate [32].

### 3.13. In Vitro Tests for the Assessment of Anti-Inflammatory Activity

#### 3.13.1. LOX Inhibition Assay

Type I-B soybean lipoxygenase is a commonly used enzyme for studying the anti-inflammatory properties of various compounds [19]. The lipoxygenase (LOX) inhibition assay measures the enzyme’s ability to convert sodium linoleate to 13-hydroperoxylinoleic acid. The inhibition of the enzyme by the investigated compounds was determined by measuring the absorbance at 234 nm for each prepared sample. Specifically, the test compound, Tris buffer, an enzyme solution (5 × 10^3^ units/mL in saline), and sodium linoleate (3 mM in Tris buffer, pH 9), were added to a UV cuvette, and the absorbance was recorded. The activities of the test compounds were compared with activity of the appropriate standard inhibitor quercetin. All measurements were performed in triplicate.

#### 3.13.2. COX Inhibition Assay

The COX-2 Inhibitor Screening Kit (Fluorometric) (Abcam limited, Cambridge, UK) is a sensitive assay for screening COX-2 inhibitors by fluorometrically detecting Prostaglandin G2, an intermediate formed by COX enzymatic activity The COX-2 inhibitory activity was evaluated following the manufacturer’s instructions [33]. Tested compounds were prepared as 5 mM DMSO stock solutions, diluted 5-fold with assay buffer, and 10 µL was used per well. Controls included celecoxib (inhibitor control), DMSO (solvent control) and enzyme control. A reaction mix containing COX-2 enzyme, probe, cofactor, and assay buffer was added (80 µL), followed by 10 µL arachidonic acid/NaOH to start the reaction. Fluorescence (Ex/Em = 535/587 nm) was monitored kinetically at 25 °C. All measurements were performed in triplicate.

### 3.14. Statistical Analysis

Antimicrobial activities (MIC and MMC values) were determined descriptively and not subjected to statistical testing. Data on antioxidant and anti-inflammatory activities are expressed as mean ± standard deviation (SD) or mean ± standard error of the mean (SE), as indicated in the tables. Differences in antioxidant activities (DPPH and ABTS assays) at varying concentrations and incubation times were assessed by one-way or multi-factorial ANOVA, followed by Tukey’s multiple range test when significant effects were observed. IC_50_ values were estimated by nonlinear regression analysis of dose–response curves. Statistical significance was set at *p* < 0.05. All analyses were conducted using SPSS Statistics software (v. 24.0, IBM Corp., Armonk, NY, USA).

## 4. Conclusions

This study provides the first comprehensive assessment of the biological activities of *B. vulgaris* metabolites and their synthetic analogues. Phenolic derivatives, particularly compounds **2** and **8**, demonstrated the most pronounced and broad-spectrum antimicrobial and antibiofilm effects, while racemic barbarin (**4**) emerged as a selective lipoxygenase inhibitor with promising anti-inflammatory potential. In contrast, all tested metabolites displayed weak COX-2 inhibition and negligible antioxidant activity, indicating that their biological effects arise from specific microbial and enzymatic interactions rather than nonspecific radical scavenging. The observed structure–activity relationships indicate that hydroxylated phenolic derivatives function as potent antimicrobial and antibiofilm agents, whereas heterocyclic scaffolds act as selective enzyme inhibitors. This multifunctional profile reflects their ecological role in plant defense and positions them as promising scaffolds for pharmacological development.

Future research should focus on detailed structure–activity relationship studies, with particular attention to stereochemical effects and substitution patterns, in order to optimize potency and selectivity. In vivo validation will be essential to confirm the pharmacological relevance suggested by our in vitro findings, while studies on synergistic combinations with conventional antimicrobials or natural metabolites may further expand their therapeutic scope. Considering their activity against phytopathogens, these compounds also hold potential for agricultural applications, bridging ecological function with potential applications in medicine and agriculture.

## Figures and Tables

**Table 1:** Antimicrobial activity of compounds **1**–**8** ^1^.

Species/Compounds	1		2		3		4		5		6		7		8	
	MIC	MMC	MIC	MMC	MIC	MMC	MIC	MMC	MIC	MMC	MIC	MMC	MIC	MMC	MIC	MMC
*B. subtilis* ATCC 6633	2.5	>5	0.078	1.25	2.5	5	2.5	5	>5	>5	>5	>5	5	5	0.312	>5
*B. cereus*	5	5	0.156	0.625	2.5	2.5	5	5	>5	>5	>5	>5	5	5	0.156	1.25
*B. pumilus* NCTC 8241	1.25	2.5	0.156	0.156	0.625	0.625	1.25	2.5	2.5	5	>5	>5	5	5	0.156	0.156
*B. animalis* subsp. *lactis*	1.25	2.5	0.078	0.625	0.625	2.5	1.25	5	1.25	5	>5	>5	2.5	5	<0.039	0.625
*S. aureus* ATCC 25923	5	5	0.156	0.156	0.625	0.625	1.25	2.5	5	>5	>5	>5	5	5	0.156	0.625
*S. aureus*	5	5	0.312	0.312	0.156	0.312	1.25	1.25	>5	>5	>5	>5	2.5	5	0.078	0.312
*E. coli* ATCC 25922	1.25	1.25	0.312	0.312	0.625	1.25	2.5	5	5	5	>5	>5	1.25	2.5	0.078	0.312
*E. coli*	2.5	5	0.312	0.312	1.25	5	2.5	5	5	5	>5	>5	1.25	2.5	<0.039	0.625
*P. mirabilis*	5	5	0.156	0.312	2.5	2.5	2.5	5	5	5	>5	>5	2.5	2.5	0.156	0.625
*S. enterica*	5	5	0.625	1.25	2.5	2.5	2.5	5	5	>5	>5	>5	1.25	2.5	0.312	1.25
*K. pneumoniae*	5	5	0.625	0.625	2.5	2.5	2.5	5	5	5	>5	>5	2.5	2.5	0.312	0.625
*C. albicans* ATCC 10231	1.25	>5	2.5	>5	0.312	1.25	5	5	5	>5	>5	>5	2.5	5	2.5	2.5
*T. viride* ATCC 13233	0.625	1.25	0.156	0.312	2.5	5	2.5	2.5	5	5	>5	>5	1.25	2.5	<0.039	<0.039
*A. flavus* ATCC 9170	1.25	2.5	0.312	0.625	5	5	>5	>5	>5	>5	>5	>5	2.5	2.5	<0.039	<0.039
*F. solani* ATCC 11712	1.25	1.25	0.156	0.234	0.625	0.625	2.5	3.75	5	>5	>5	>5	2.5	2.5	<0.039	<0.039

^1^ Data are given as mg/mL; MIC—minimum inhibitory concentration; MMC—minimum microbicidal concentration (encompassing MBC or MFC); “>5” indicates no activity at the highest tested concentration (5 mg/mL).

**Table 2:** Antimicrobial activity of tested antibiotics/antimycotic ^1^.

Species/Antibiotics/ Antimycotic	Doxycycline		Ampicillin		Ketoconazole		Amphotericin B	
	MIC	MMC	MIC	MMC	MIC	MMC	MIC	MMC
*B. subtilis* ATCC 6633	1.953	31.25	3	4	-	-	-	-
*B. cereus*	0.977	7.81	4	6	-	-	-	-
*B. pumilus* NCTC 8241	-	-	8	12	-	-	-	-
*B. animalis* subsp. *lactis*	4	8	<0.06	0.12	-	-	-	-
*S. aureus* ATCC 25923	0.224	3.75	0.25	0.75	-	-	-	-
*S. aureus*	0.45	7.81	<0.06	<0.06	-	-	-	-
*E. coli* ATCC 25922	15.63	31.25	0.37	0.5	-	-	-	-
*E. coli*	15.63	62.5	1.2	2.1	-	-	-	-
*P. mirabilis*	15.63	62.5	>128	>128	-	-	-	-
*S. enterica*	15.63	31.25	1	1	-	-	-	-
*K. pneumoniae*	2	32	>128	>128	-	-	-	-
*C. albicans* ATCC 10231	-	-	-	-	1.96	1.96	<0.098	<0.098
*T. viride* ATCC 13233	-	-	-	-	62.5	125	0.78	1.56
*A. flavus* ATCC 9170	-	-	-	-	<0.49	1.96	0.39	0.78
*F. solani* ATCC 11712	-	-	-	-	0.08	0.156	-	-

^1^ Data given as µg/mL; MIC—minimum inhibitory concentration; MMC—minimum microbiocidal concentration (encompassing MBC or MFC); “-”—not tested.

**Table 3:** Antibiofilm activity of selected compounds ^1^.

Species/Compounds	2		3		4		7		8		Tetracycline	
	BIC_50_	BIC_90_	BIC_50_	BIC_90_	BIC_50_	BIC_90_	BIC_50_	BIC_90_	BIC_50_	BIC_90_	BIC_50_	BIC_90_
*B. subtilis* ATCC 6633	290	1250	1190	2500	2700	>5000	>5000	>5000	1100	2300	˂15.6	15.6
*S. aureus* ATCC 25923	220	2120	2500	>5000	1500	>5000	>5000	>5000	868	1360	250	300
*P. mirabilis*	1158	4500	3420	>5000	3170	>5000	3100	4754	1428	2400	320	>1000
*S. enterica*	1100	3200	2400	>5000	1000	4470	1254	>5000	1547	>5000	˂15.6	180
*K. pneumoniae*	580	3600	1262	>5000	3978	>5000	4400	>5000	570	2000	6	125

^1^ Data given as µg/mL; BIC_50_—biofilm inhibitory concentration required to reduce biofilm coverage by 50%; BIC_90_—biofilm inhibitory concentration required to reduce biofilm coverage by 90%; “>5000” indicates no activity at the highest tested concentration (5000 µg/mL).

**Table 4:** The results of the DPPH test of **1**–**8** and standard compound ^1^.

Compound	DPPH Scavenging Ability (%) ^1^					
	25 µM		50 µM		100 µM	
	20 min	60 min	20 min	60 min	20 min	60 min
**1**	1.6 ± 1.2	1.8 ± 1.5	2.5 ± 1.1	2.4 ± 1.4	2.6 ± 1.2	2.9 ± 1.7
**2**	11.9 ± 0.7	12.0 ± 0.4	15.6 ± 0.3	15.7 ± 1.4	24.9 ± 1.1	25.2 ± 1.6
**3**	1.0 ± 0.3	1.1 ± 0.5	1.9 ± 0.8	2.1 ± 1.1	7.8 ± 1.8	7.9 ± 1.7
**4**	1.2 ± 0.7	1.3 ± 0.5	1.7 ± 0.8	2.0 ± 0.9	3.8 ± 1.2	3.5 ± 1.7
**5**	7.0 ± 0.9	8.4 ± 0.5	14.8 ± 0.7	16.4 ± 0.4	21.6 ± 1.2	22.4 ± 0.7
**6**	3.1 ± 0.6	3.0 ± 0.7	4.2 ± 0.8	4.6 ± 0.6	5.9 ± 0.9	5.8 ± 1.4
**7**	1.1 ± 0.4	0.9 ± 0.7	1.5 ± 0.9	1.5 ± 0.8	2.6 ± 1.0	2.7 ± 1.5
**8**	4.4 ± 1.0	4.7 ± 1.2	7.3 ± 1.3	8.1 ± 1.3	13.1 ± 0.7	14.2 ± 0.9
Quercetin	95.3 ± 0.8	95.1 ± 0.9	96.8 ± 1.0	96.5 ± 0.9	95.1 ± 0.9	95.4 ± 0.8

^1^ Results represent mean values ± standard deviation (SD) of three independent measurements.

**Table 5:** The results of the ABTS scavenging activity of **1**–**8** and corresponding referent compound ^1^.

Compound	ABTS Scavenging Ability (%) ^1^		
	25 µM	50 µM	100 µM
**1**	1.7 ± 1.1	2.2 ± 1.2	2.6 ± 0.8
**2**	7.6 ± 1.0	13.4± 0.9	13.4 ± 0.8
**3**	1.2 ± 0.3	1.9 ± 1.2	2.6 ± 0.7
**4**	0.9 ± 0.1	1.6 ± 0.9	2.5 ± 0.3
**5**	6.6 ± 0.2	12.3 ± 0.8	19.1 ± 0.6
**6**	2.6 ± 0.7	3.9 ± 1.2	5.1 ± 0.9
**7**	1.3 ± 0.1	2.7 ± 1.0	3.9 ± 0.4
**8**	2.9 ± 1.3	6.8 ± 1.1	11.9 ± 0.8
Trolox	97.5 ± 0.2	99.3 ± 0.1	99.5 ± 0.3

^1^ Results represent mean values ± standard deviation (SD) of three independent measurements.

**Table 6:** In vitro LOX and COX-2 inhibition results of compounds **1**–**8** and the corresponding reference compound.

Compound	Percentage and IC_50_ of LOX Inhibition ^1^		Percentage of COX-2 Inhibition ^2^	
	100 µM	IC_50_ (µM)	100 µM	10 µM
**1**	47.5 ± 1.5	125.5 ± 0.4	-	-
**2**	55.5 ± 0.1	97.4 ± 1.3	-	-
**3**	23.8 ± 0.8	n.d.	-	-
**4**	90.5 ± 0.1	61.6 ± 0.6	14.23 ± 1.0	4.50 ± 1.6
**5**	40.0 ± 0.5	122.5 ± 1.5	21.25 ± 1.3	8.11 ± 1.0
**6**	34.6 ± 0.4	n.d.	-	-
**7**	18.3 ± 0.3	n.d.	-	-
**8**	21.2 ± 0.3	n.d.	-	-
Quercetin	92.0 ± 0.9	43.2 ± 0.5	-	-

^1^ Results represent mean values ± standard deviation (SD) of three independent measurements. ^2^ Results represent mean values ± standard error (SE) of three independent measurements; “-”—not tested.

## Data Availability

Data are contained within the article and Supplementary Materials.

## Supplementary Materials

The following supporting information can be downloaded at: https://www.mdpi.com/article/10.3390/molecules30234606/s1, Synthetic and Isolation procedures, Figures S1–S33. References [34,35,36,37,38,39,40] are cited in the Supplementary Materials.
