# Supplementary material for: Glucosinolate-Derived Metabolites from Barbarea vulgaris (Brassicaceae): Evaluation of Antimicrobial, Antioxidant, and Anti-Inflammatory Potentials
Source: Molecules. 2025 Nov 30;30(23):4606. doi: 10.3390/molecules30234606 (PMC12693701; doi:10.3390/molecules30234606)
Supplement: Supplementary file 1 [file molecules-30-04606-s001.zip › molecules-3957815-supplementary.pdf]

Supplementary Material

for

Glucosinolate-Derived Metabolites from *Barbarea vulgaris* (Brassicaceae): Evaluation of Antimicrobial, Antioxidant, and Anti-Inflammatory Potentials

Elvira Mavric-Scholze [1], Amina Gusinac [2], Milan Dekić [3,*], Ivan Palić [2], Edina Avdović [4], Dušica Simijonović [4], Mirjana Grujović [4], Katarina Marković [4], Vladimir Dobričić [5], Jelena Bošković [5], Zoran Marković [3], and Niko Radulović [2]

[1] Anhalt University of Applied Sciences, Department for Applied Biosciences and Process Engineering, Bernburger Str. 55, Köthen 06366, Germany; elvira.mavric-scholze@hs-anhalt.de

[2] Department of Chemistry, Faculty of Sciences and Mathematics, University of Niš, Višegradska 33, 18106 Niš, Serbia; aminag5667@gmail.com (A.G.); ivanpalic@yahoo.com (I.P.); nikoradulovic@yahoo.com (N.R.)

[3] Department of Sciences and Mathematics, State University of Novi Pazar, Vuka Karadžića 9, 36300 Novi Pazar, Serbia; mdekic@np.ac.rs (M.D.); zmarkovic@uni.kg.ac.rs (Z.M.)

[4] Institute for Information Technologies, University of Kragujevac, Jovana Cvijića bb, 34000 Kragujevac, Serbia; edina.avdovic@pmf.kg.ac.rs (E.A.); dusica.simijonovic@uni.kg.ac.rs (D.S.); mirjana.grujovic@pmf.kg.ac.rs (M.G.); katarina.mladenovic@pmf.kg.ac.rs (K.M.)

[5] Department of Pharmaceutical Chemistry, University of Belgrade-Faculty of Pharmacy, Vojvode Stepe 450, 11221 Belgrade, Serbia; vladimir.dobricic@pharmacy.bg.ac.rs (V.D.); jelena.boskovic@pharmacy.bg.ac.rs (J.B.)

* Correspondence: mdekic@np.ac.rs; Tel.: +38169660333

Table of Contents

Synthetic route, spectral data, and additional analytical details of compounds 1–4……… 3

Nasturlexin A (**1**)……… 3

Fig. S1. Synthetic scheme leading to nasturlexin A (**1**)……… 3

Methyl-4-hydroxyphenylethyl dithiocarbamate (**2**)……… 3

Fig. S2. Synthetic scheme leading to methyl-4-hydroxyphenylethyl dithiocarbamate (**2**)……… 3

Raphanusamic acid (**3**)……… 3

Fig. S3. Synthetic scheme leading to raphanusamic acid (**3**)……… 3

(±)-Barbarin (**4**)……… 4

Fig. S4. Synthetic scheme leading to (±)-barbarin (**4**)……… 4

Experimental spectra of compounds 1–4 ……… 5

Fig. S5 ^{1}H NMR spectrum of nasturlexin A (methyl phenethylcarbamodithioate) (**1**) ($CDCl_3$, 400 MHz, 20 °C)……… 5

Fig. S6 ^{13}C NMR spectrum of nasturlexin A (methyl phenethylcarbamodithioate) (**1**) ($CDCl_3$, 100.6 MHz, 20 °C)……… 5

Fig. S7. DEPT90 (up) and DEPT135 NMR spectra (down) of nasturlexin A (methyl phenethylcarbamodithioate) (**1**)… 6

Fig. S8 HSQC (up) and HMBC spectra (down) of nasturlexin A (methyl phenethylcarbamodithioate) (**1**)……… 7

Fig. S9 ^{1}H–^{1}H COSY (up) and NOESY spectra (down) of nasturlexin A (methyl phenethylcarbamodithioate) (**1**)……… 8

Fig. S10 EIMS (up) and IR spectra (down) of nasturlexin A (methyl phenethylcarbamodithioate) (**1**)……… 9

Fig. S11 ^{1}H NMR spectrum of methyl 4-hydroxyphenethylcarbamodithioate (**2**) ($CDCl_3$, 400 MHz, 20 °C)……… 10

Fig. S12 ^{13}C NMR spectrum of methyl 4-hydroxyphenethylcarbamodithioate (**2**) ($CDCl_3$, 100.6 MHz, 20 °C)……… 10

Fig. S13 HSQC (up) and HMBC spectra (down) of methyl 4-hydroxyphenethylcarbamodithioate (**2**)………….………. 11

Fig. S14 ^{1}H–^{1}H COSY (up) and NOESY spectra (down) of methyl 4-hydroxyphenethylcarbamodithioate (**2**)………… 12

Fig. S15 IR spectrum (down) of methyl 4-hydroxyphenethylcarbamodithioate (**2**)……………………………………… 13

Fig. S16 ^{1}H NMR spectrum of 2-thioxothiazolidine-4-carboxylic acid (**3**) ($CDCl_3$, 400 MHz, 20 °C)…………………….. 14

Fig. S17 ^{13}C NMR spectrum of 2-thioxothiazolidine-4-carboxylic acid (**3**) ($CDCl_3$, 100.6 MHz, 20 °C)………………….. 15

Fig. S18 IR spectrum of 2-thioxothiazolidine-4-carboxylic acid (**3**)………………………………………………………. 15

Fig. S19 EIMS spectrum of 2-thioxothiazolidine-4-carboxylic acid (**3**)……………………………………………………… 16

Fig. S20 ^{1}H NMR of *rac*-5-phenyl-1,3-oxazolidine-2-thione (*rac*-barbarin, **4**) ($CDCl_3$, 400 MHz, 20 °C)………………… 17

Fig. S21 EIMS spectrum of *rac*-barbarin (**4**)……………………………………………………………………………… 17

Experimental procedures, characterization data and spectra of compounds 5–8…………………………………….……. 18

Isolation of (*S*)-5-phenyl-1,3-oxazolidine-2-thione ((*S*)-barbarin, **5**)……………………………………………………. 18

Determination of absolute configuration of **5** via NMR titration using a chiral lanthanide shift reagent…..………………… 18

Separation of ^{1}H NMR signals of barbarin enantiomers using a chiral lanthanide shift reagent ………….……………… 18

Fig. S22 ^{1}H NMR spectrum of (*S*)-5-phenyl-1,3-oxazolidine-2-thione ((*S*)-barbarin) (**5**) ($CDCl_3$, 400 MHz, 20 °C)…..... 19

Fig. S23 ^{13}C NMR spectrum of (*S*)-5-phenyl-1,3-oxazolidine-2-thione ((*S*)-barbarin) (**5**) ($CDCl_3$, 100.6 MHz, 20 °C).... 19

Fig. S24 Partial ^{1}H NMR spectra showing the chemical shift changes of the C-4a, C-4b, and C-5 proton signals upon incremental additions of Eu(hfc)$_3$ to a racemic mixture of barbarin, recorded in C_6D_6 at 400 MHz……………………… 20

Fig. 25. A) The ^{1}H NMR signal shifts after incremental additions of Eu(hfc)$_3$ to isolated (*S*)-barbarin recorded in C_6D_6 (400 MHz). **B)** Relationship between δ_H values (ppm) and molar Eu(hfc)$_3$/(*S*)-barbarin ratio for H-4a, H-4b, and H-5 protons of (*S*)-barbarin with corresponding equations of the regression lines……………………………………….. 20

Isolation of 5-phenyl-1,3-thiazolidin-2-one (**6**)………………………………………………….. …………………………… 21

Fig. S26 ^{1}H NMR spectrum of 5-phenyl-1,3-thiazolidin-2-one (**6**) (DMSO$_{d6}$, 400 MHz, 20 °C)……..…………………….. 22

Fig. S27 ^{13}C NMR spectrum of 5-phenyl-1,3-thiazolidin-2-one (**6**) (DMSO$_{d6}$, 100.6 MHz, 20 °C)……………………….. 22

Synthesis of (±)-5-phenyl-1,3-oxazolidin-2-one (resedine, **7**)…………………………………………………………..……. 23

Fig. S28 Synthetic scheme leading to the (±)-5-phenyl-1,3-oxazolidin-2-one (**7**)……………….……………………… 23

Fig. S29 ^{1}H NMR spectrum of (±)-5-phenyl-1,3-oxazolidin-2-one (resedine) (**7**) ($CDCl_3$, 400 MHz, 20 °C)……………. 24

Fig. S30 ^{13}C NMR spectrum of (±)-5-phenyl-1,3-oxazolidin-2-one (resedine) (**7**) ($CDCl_3$, 100.6 MHz, 20 °C)……..….. 24

Synthesis of 2-(4-hydroxyphenyl)ethyl isothiocyanate (**8**)……………………………………………………………………. 25

Fig. S31 Synthetic scheme leading to the 2-(4-hydroxyphenyl)ethyl isothiocyanate (**8**)……………………………..…. 25

Fig. S32 ^{1}H NMR spectrum of 2-(4-hydroxyphenyl)ethyl isothiocyanate (**8**) ($CDCl_3$, 400 MHz, 20 °C)…………………. 26

Fig. S33 ^{13}C NMR spectrum of 2-(4-hydroxyphenyl)ethyl isothiocyanate (**8**) ($CDCl_3$, 100.6 MHz, 20 °C)………………. 26

Synthetic route, spectral data, and additional analytical details of compounds 1–4

Nasturlexin A (1). White solid. Yield: 78%. MP 52–53 °C (lit. 51–52 °C). FTIR (neat), cm^{-1}: 3320 (N–H), 2936 (C–H), 1598 (arC–C), 1500 (arC-C), 1453, 1381, 1338, 1297, 1199, 1090, 1061, 1090, 940, 772, 700. ^{1}H NMR ($CDCl_3$), δ: 2.61 (s, 3H, $C\underline{H_3}$), 2.97 (t, 2H, J = 7.0, H-7), 3.99 (m, 2H, H-8), 6.95 (brs, 1H, NH), 7.20–7.29 (m, 3H, H-2, H-4, H-6), 7.30–7.36 (m, 2H, H-3, H-5), and signals due to a minor rotamer (ca. 24%) at 2.68 (s, 3H, CH_3), 2.97 (t, 2H, J = 7.0, H-7), 3.71 (m, 2H, H-8), 7.20–7.29 (m, 3H, H-2, H-4, H-6), 7.30–7.36 (m, 2H, H-3, H-5), 7.69 (brs, 1H, NH). ^{13}C NMR ($CDCl_3$), δ: 18.1 ($\underline{C}H_3$), 34.2 (C-7), 48.0 (C-8), 126.9 (C-4), 128.7 (C-2, C-6, two overlapping signals), 128.8 (C-3, C-5, two overlapping signals), 138.1 (C-1), 199.1 (C=S), and signals due to a minor rotamer at 18.9 ($\underline{C}H_3$), 34.8 (C-7), 47.3 (C-8), 126.9 (C-4), 128.7 (C-2, C-6, two overlapping signals), 128.8 (C-3, C-5, two overlapping signals), 137.2 (C-1), 201.9 (C=S). EIMS, m/z (rel. int.): 211 (24, $M^{+\bullet}$), 163 (18), 105 (27), 104 (29), 91 (100), 77 (28), 72 (41), 65 (32), 51 (23), 39 (18).

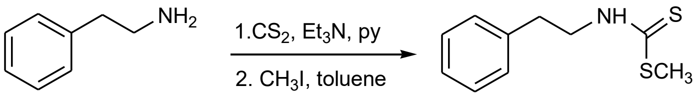

Phenethylamine

1

Fig. S1. Synthetic scheme leading to nasturlexin A (**1**)

Methyl-4-hydroxyphenylethyl dithiocarbamate (2). Pale-yellow solid. Yield: 80%. FTIR (neat), cm^{-1}: 3283 (O–H, N–H), 3024 (arC–H), 2938 (C–H), 1612, 1600, 1500, 1441, 1333, 1244, 1202, 1169, 936, 822. ^{1}H NMR ($CDCl_3$), δ: 2.61 (s, 3H, $C\underline{H_3}$), 2.90 (m, 2H, H-7), 3.96 (m, 2H, H-8), 6.92 (brs, 1H, NH), 6.78–6.82 (m, 2H, H-3, H-5), 7.05–7.11 (m, 2H, H-2, H-6), and signals due to a minor rotamer (ca. 33%) at 2.69 (s, 3H, CH_3), 2.90 (m, 2H, H-7), 3.68 (m, 2H, H-8), 6.78–6.82 (m, 2H, H-3, H-5), 7.05–7.11 (m, 2H, H-2, H-6), 7.63 (brs, 1H, NH). OH signal was not observed in ^{1}H NMR ($CDCl_3$) due to exchange broadening. ^{13}C NMR ($CDCl_3$), δ: 18.1 ($\underline{C}H_3$), 33.3 (C-7), 48.2 (C-8), 115.7 (C-3, C-5, two overlapping signals), 129.9 (C-2, C-6, two overlapping signals), 130.2 (C-1), 154.4 (C-4), 199.1 (C=S), and signals due to a minor rotamer at 18.9 ($\underline{C}H_3$), 33.9 (C-7), 47.4 (C-8), 115.5 (C-3, C-5, two overlapping signals), 130.1 (C-2, C-6, two overlapping signals), 130.2 (C-1), 154.4 (C-4), and 201.9 (C=S).

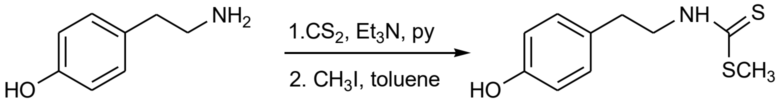

Tyramine

2

Fig. S2. Synthetic scheme leading to methyl-4-hydroxyphenylethyl dithiocarbamate (**2**)

Raphanusamic acid (3). White solid. Yield: 29%. FTIR (neat), cm^{-1}: 3337 (N-H), 1732 (C=O), 1465 (O-H), 1134, 1257 (C-O), 1203, 1172 (C=S), 1045, 961, 938, 824, 736, 782, 680 (C-S). ^{1}H NMR ($CDCl_3$), δ: 3.60 (dd, 1H, J = 11.5, 3.9, H-5), 3.86 (dd, 1H, J = 11.5, 9.3, H-5), 4.84 (ddd, 1H, 9.3, 3.9, 1.5, H-4), 10.43 (brs, 1H, $N\underline{H}$), 13.46 (brs, 1H, $COO\underline{H}$). ^{13}C NMR ($CDCl_3$), δ: 35.4 (C-5), 63.7 (C-4), 170.9 ($\underline{C}OOH$), 199.2 (C=S). EIMS, m/z (rel. int.): 163 (100, $M^{+\bullet}$), 45 (89), 59 (58), 64 (45), 58 (44), 118 (36), 76 (35), 41 (29), 77 (27), 44 (22).

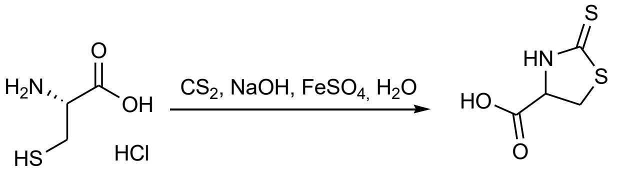

L-Cysteine hydrochloride

3

Fig. S3. Synthetic scheme leading to raphanusamic acid (**3**)

(±)-Barbarin (4). Off-white solid. MP 116–118 °C. Yield: 43%. ^{1}H NMR ($CDCl_3$), δ: 3.75 (ddd, 1H, J = 10.0, 8.1, 0.8, H-4), 4.16 (ddd, 1H, J = 10.0, 9.2, 0.8, H-4), 5.90 (dd, 1H, J = 9.2, 8.1, H-5), 7.36 – 7.44 (m, 5H, Ar-H), 7.62 (brs, 1H, N–H). EIMS, m/z (rel. int.): 179 (95, $M^{+\bullet}$), 123 (30), 119 (27), 118 (77), 107 (49), 92 (42), 91 (100), 79 (33), 77 (53), 51 (40).

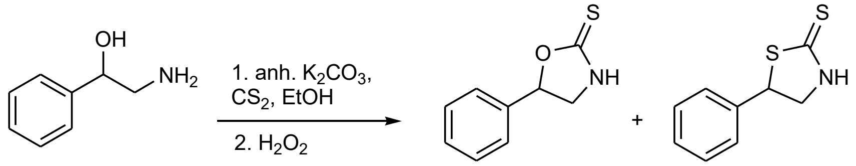

rac-2-Amino-1-phenylethanol **4** side product

Fig. S4. Synthetic scheme leading to (±)-barbarin (**4**)

Experimental spectra of compounds 1–4

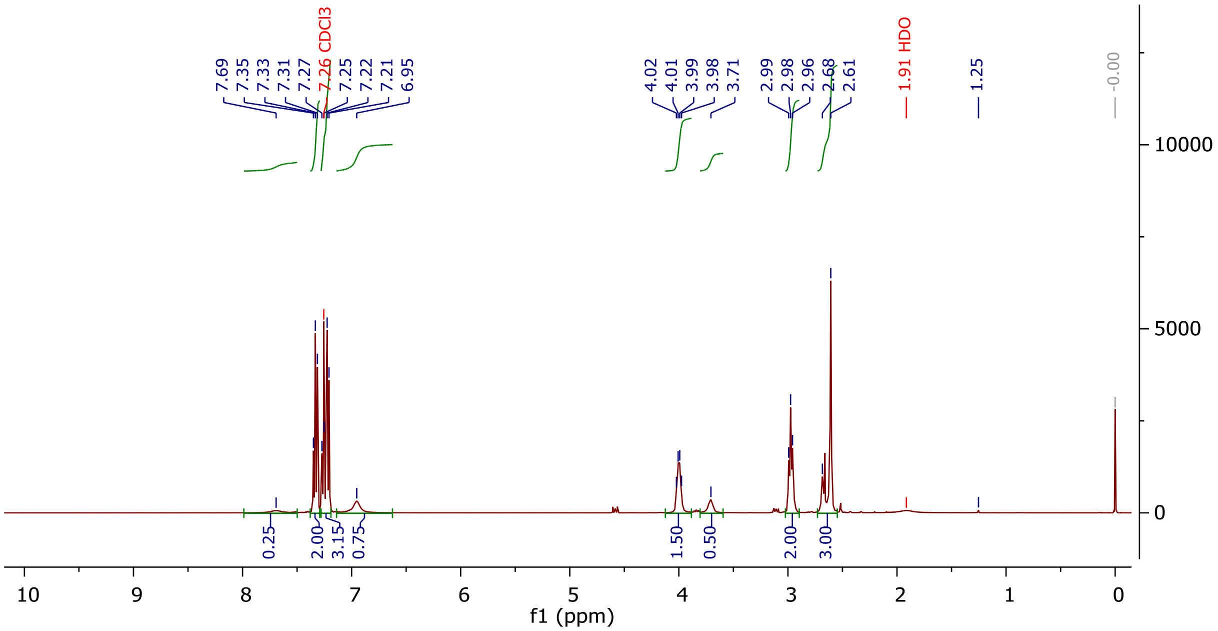

Fig. S5 ^{1}H NMR spectrum of nasturlexin A (methyl phenethylcarbamodithioate) (**1**) ($CDCl_3$, 400 MHz, 20 °C).

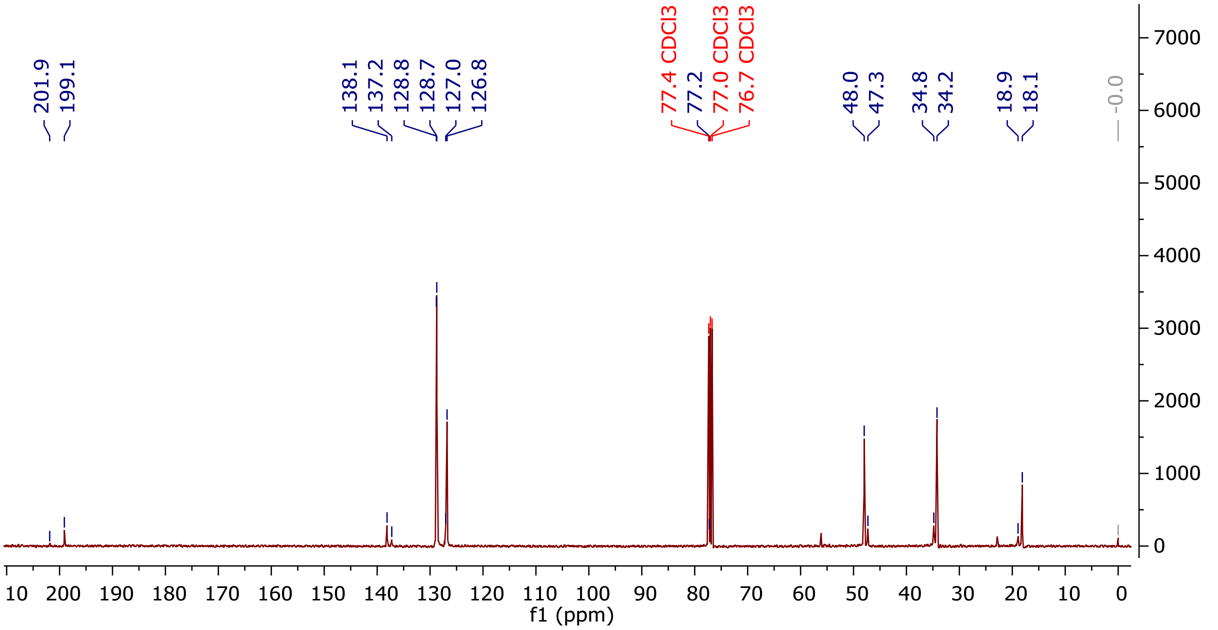

Fig. S6 ^{13}C NMR spectrum of nasturlexin A (methyl phenethylcarbamodithioate) (**1**) ($CDCl_3$, 100.6 MHz, 20 °C).

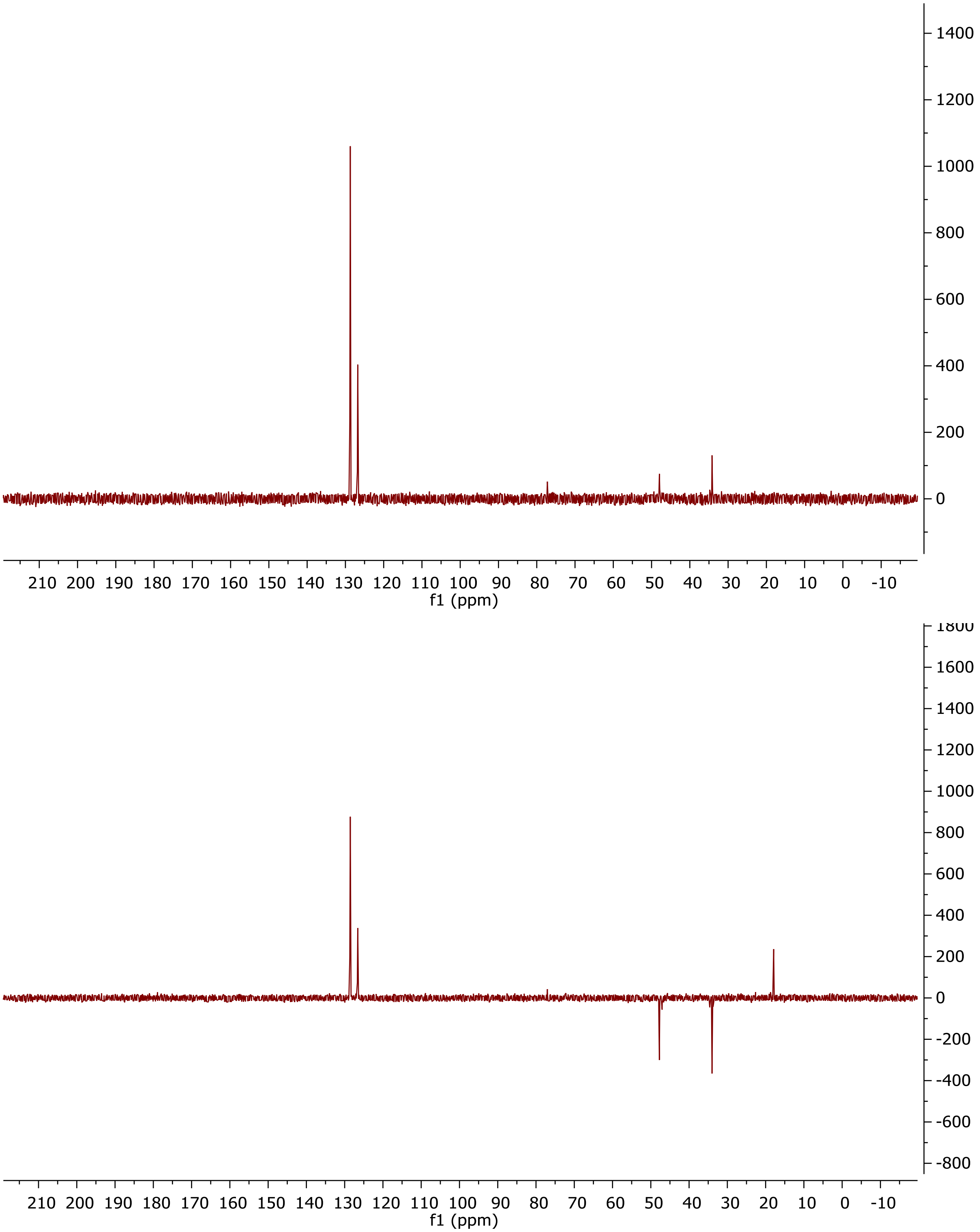

Fig. S7. DEPT90 (up) and DEPT135 NMR spectra (down) of nasturlexin A (methyl phenethylcarbamodithioate) (**1**)

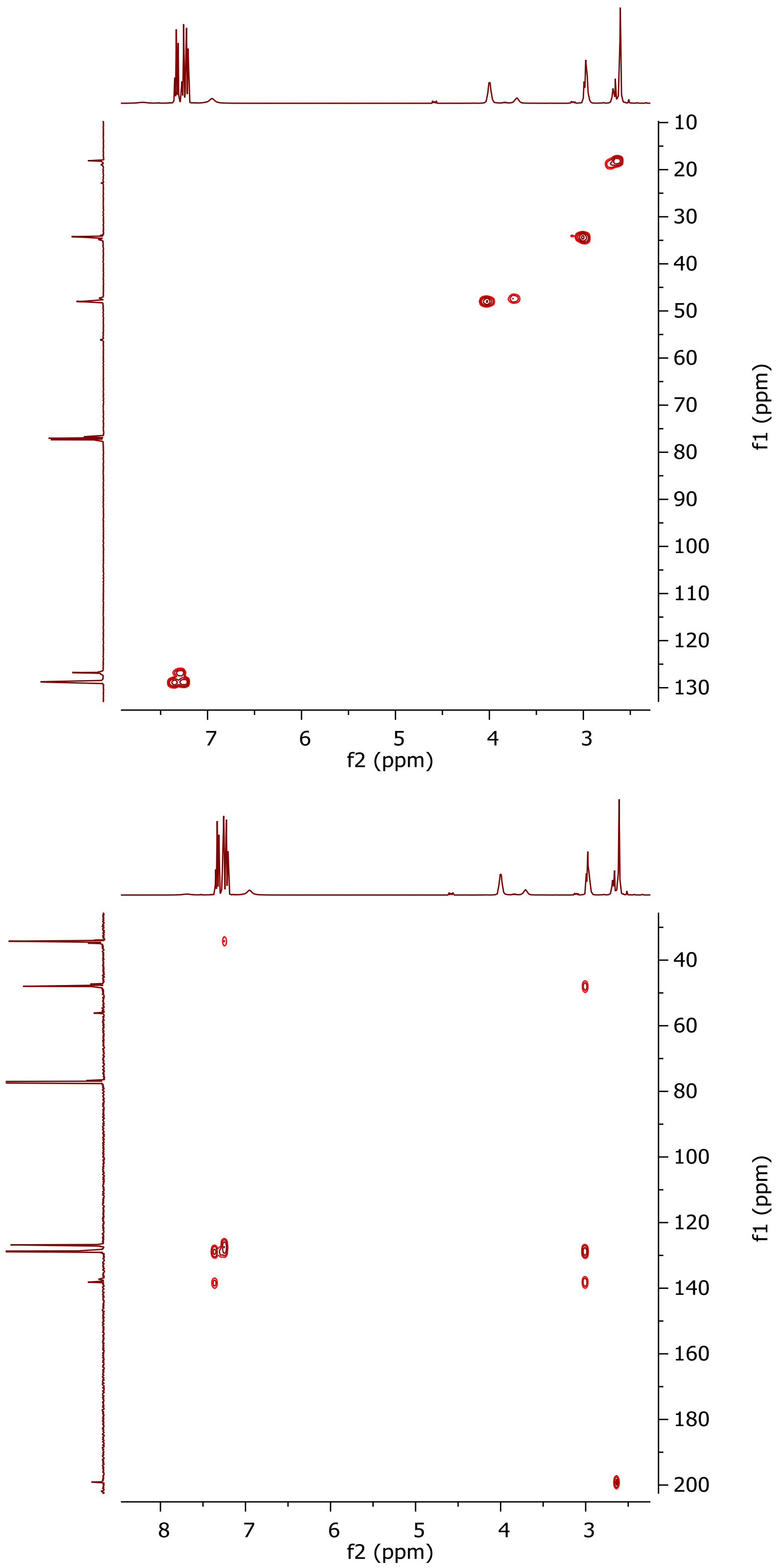

Fig. S8 HSQC (up) and HMBC spectra (down) of nasturlexin A (methyl phenethylcarbamodithioate) (**1**)

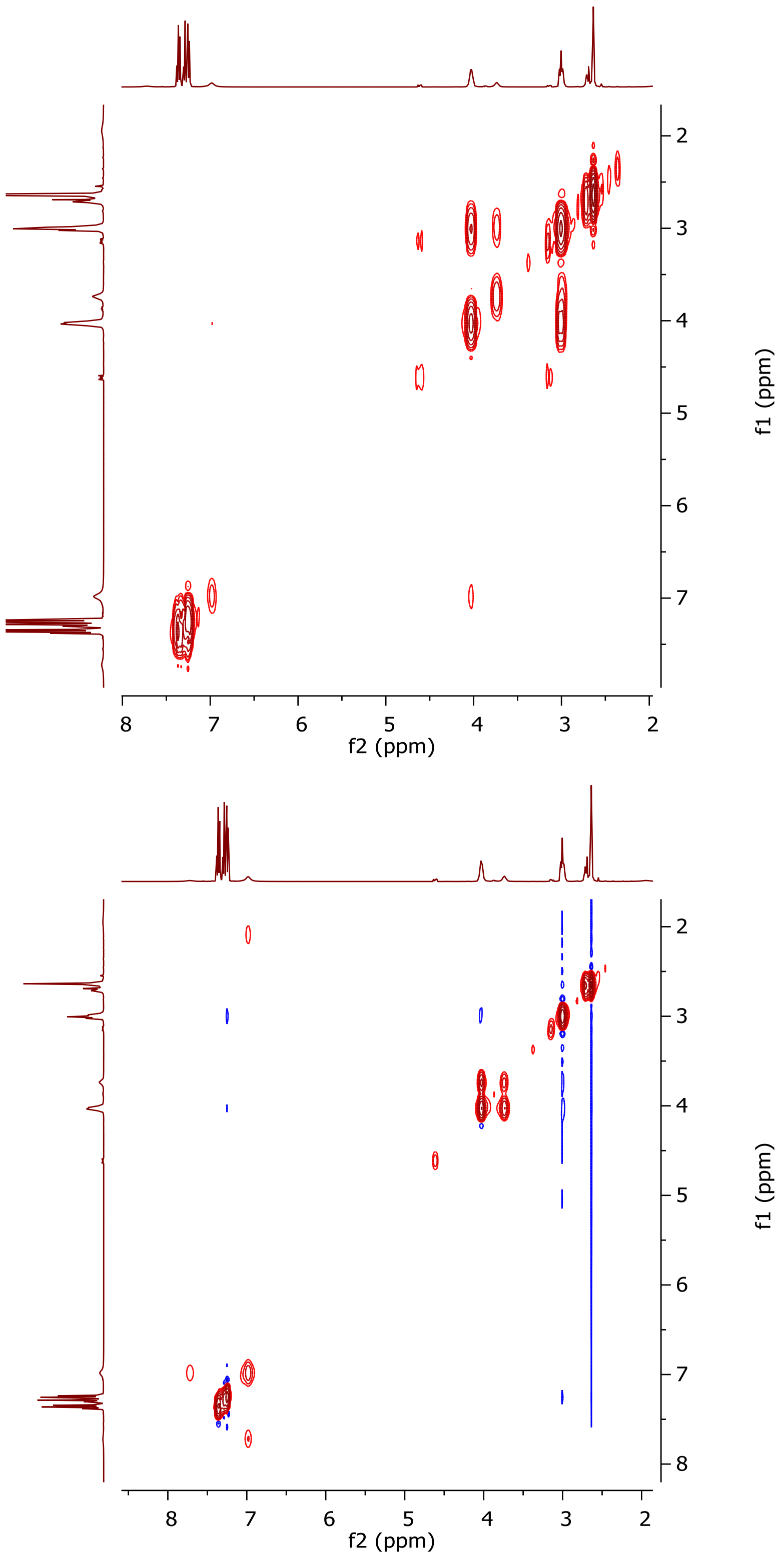

Fig. S9 ^{1}H–^{1}H COSY (up) and NOESY spectra (down) of nasturlexin A (methyl phenethylcarbamodithioate) (**1**)

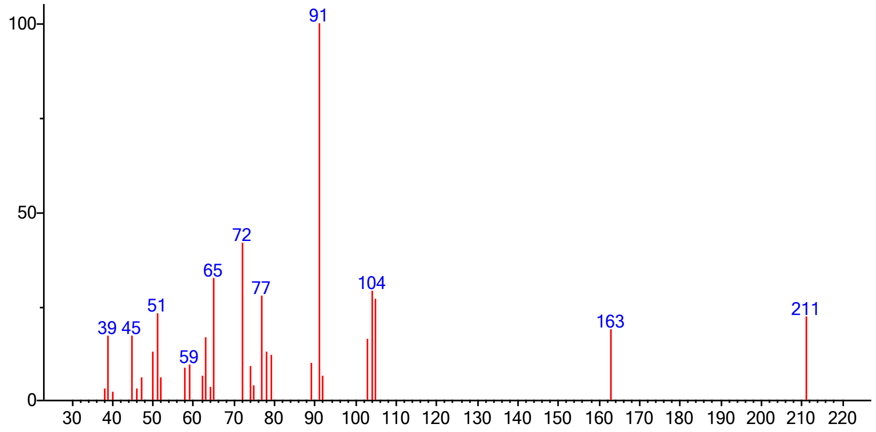

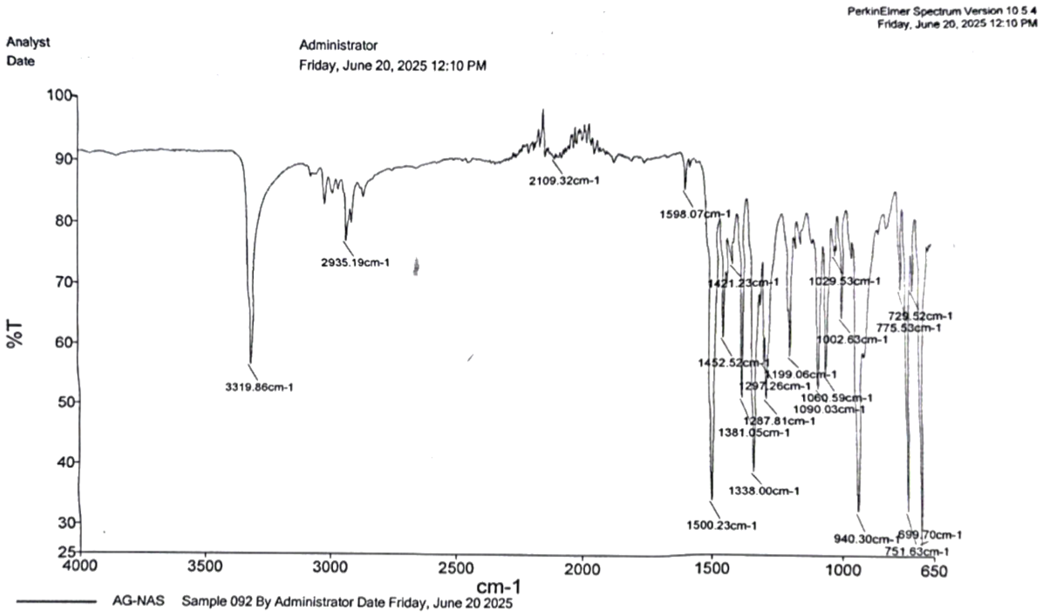

Fig. S10 EIMS (up) and IR spectra (down) of nasturlexin A (methyl phenethylcarbamodithioate) (**1**)

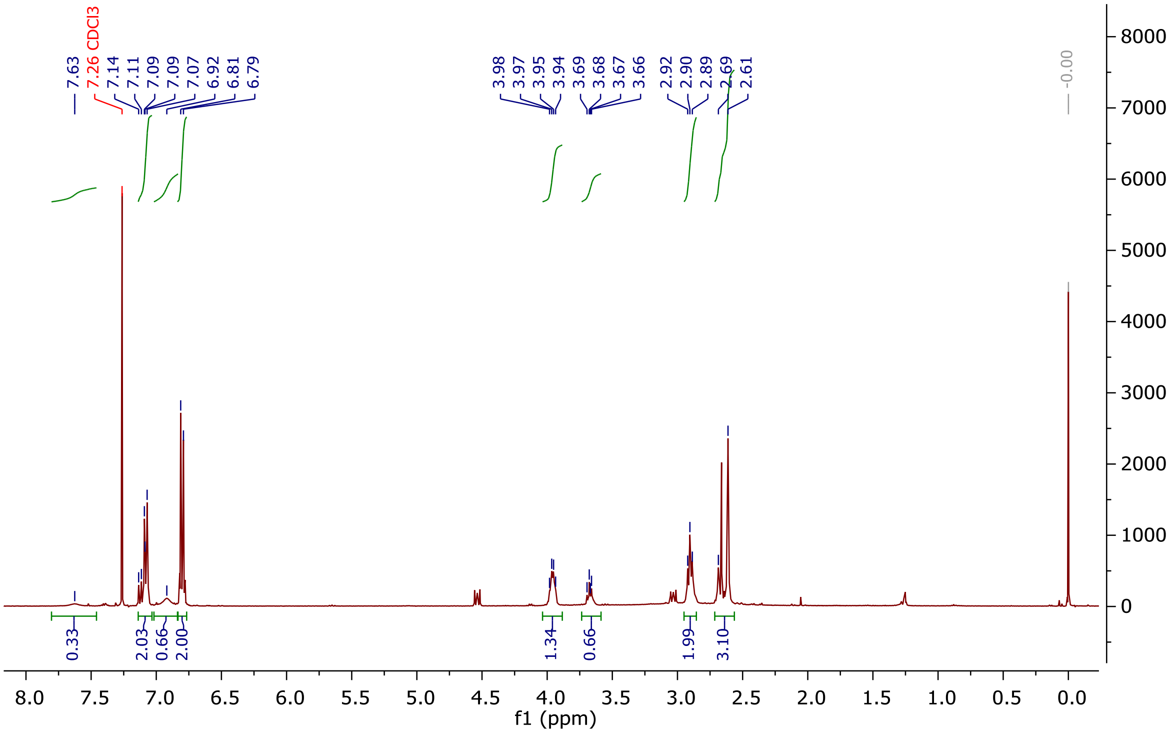

Fig. S11 ^{1}H NMR spectrum of methyl 4-hydroxyphenethylcarbamodithioate (**2**) ($CDCl_3$, 400 MHz, 20 °C).

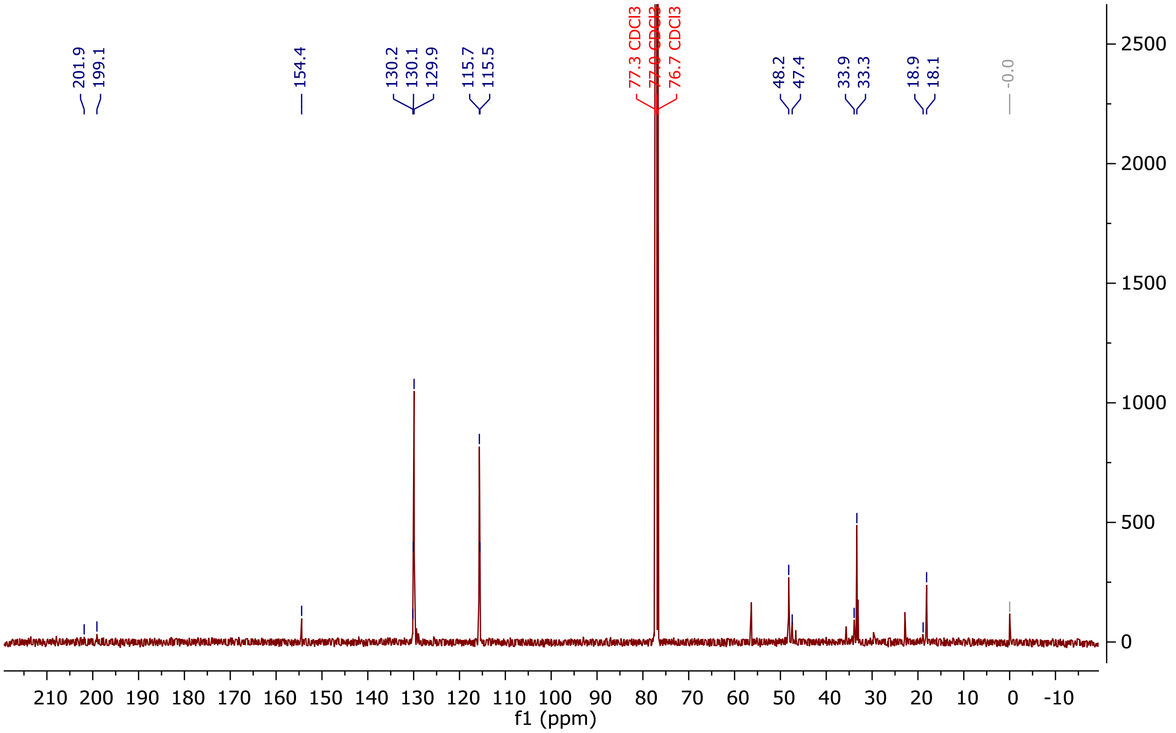

Fig. S12 ^{13}C NMR spectrum of methyl 4-hydroxyphenethylcarbamodithioate (**2**) ($CDCl_3$, 100.6 MHz, 20 °C).

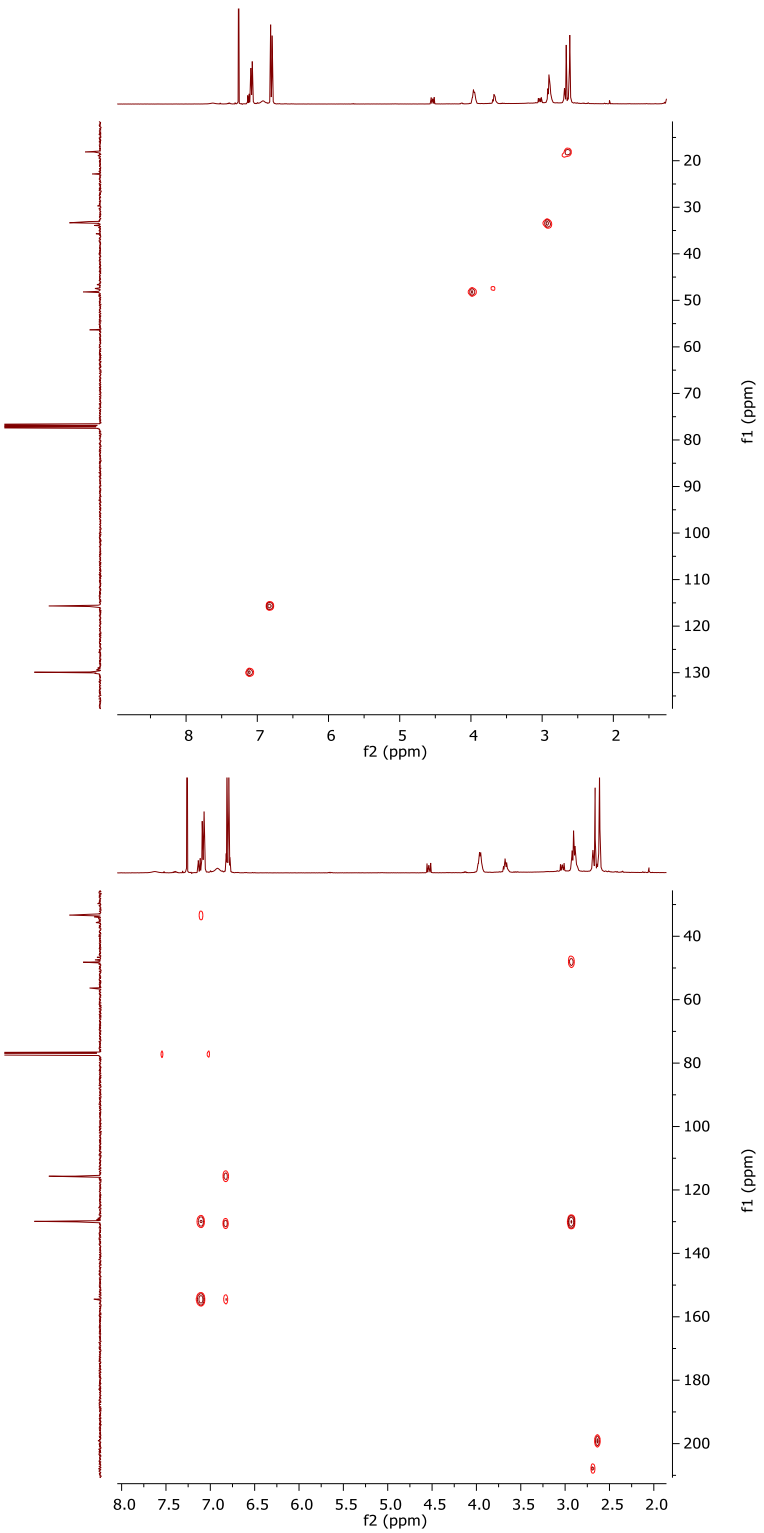

Fig. S13 HSQC (up) and HMBC spectra (down) of methyl 4-hydroxyphenethylcarbamodithioate (**2**)

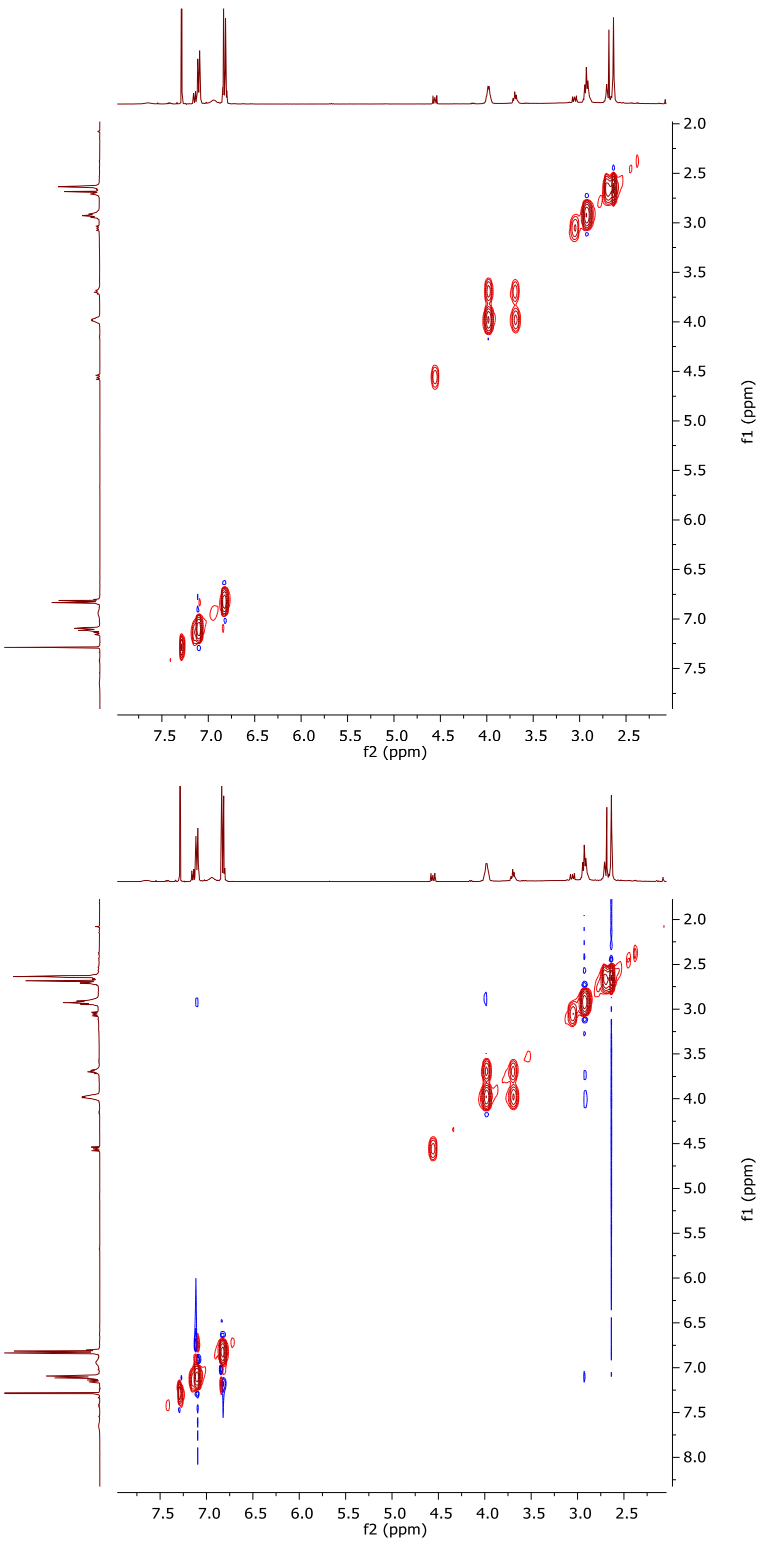

Fig. S14 ^{1}H–^{1}H COSY (up) and NOESY spectra (down) of methyl 4-hydroxyphenethylcarbamodithioate (**2**)

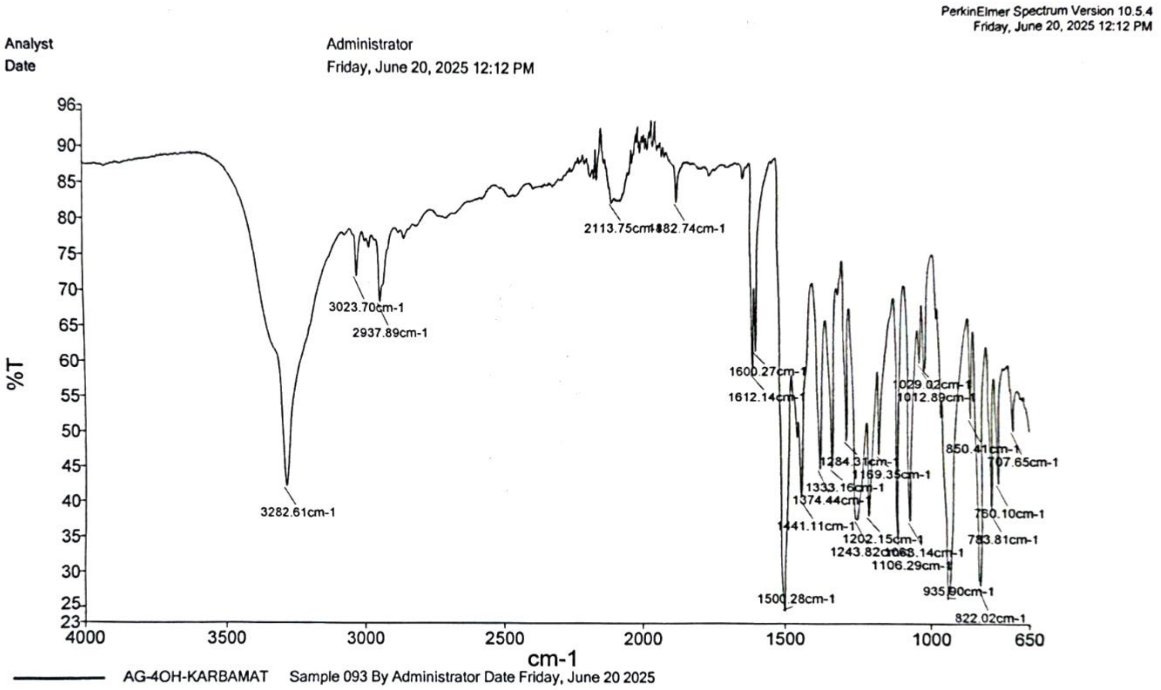

Fig. S15 IR spectrum (down) of methyl 4-hydroxyphenethylcarbamodithioate (**2**)

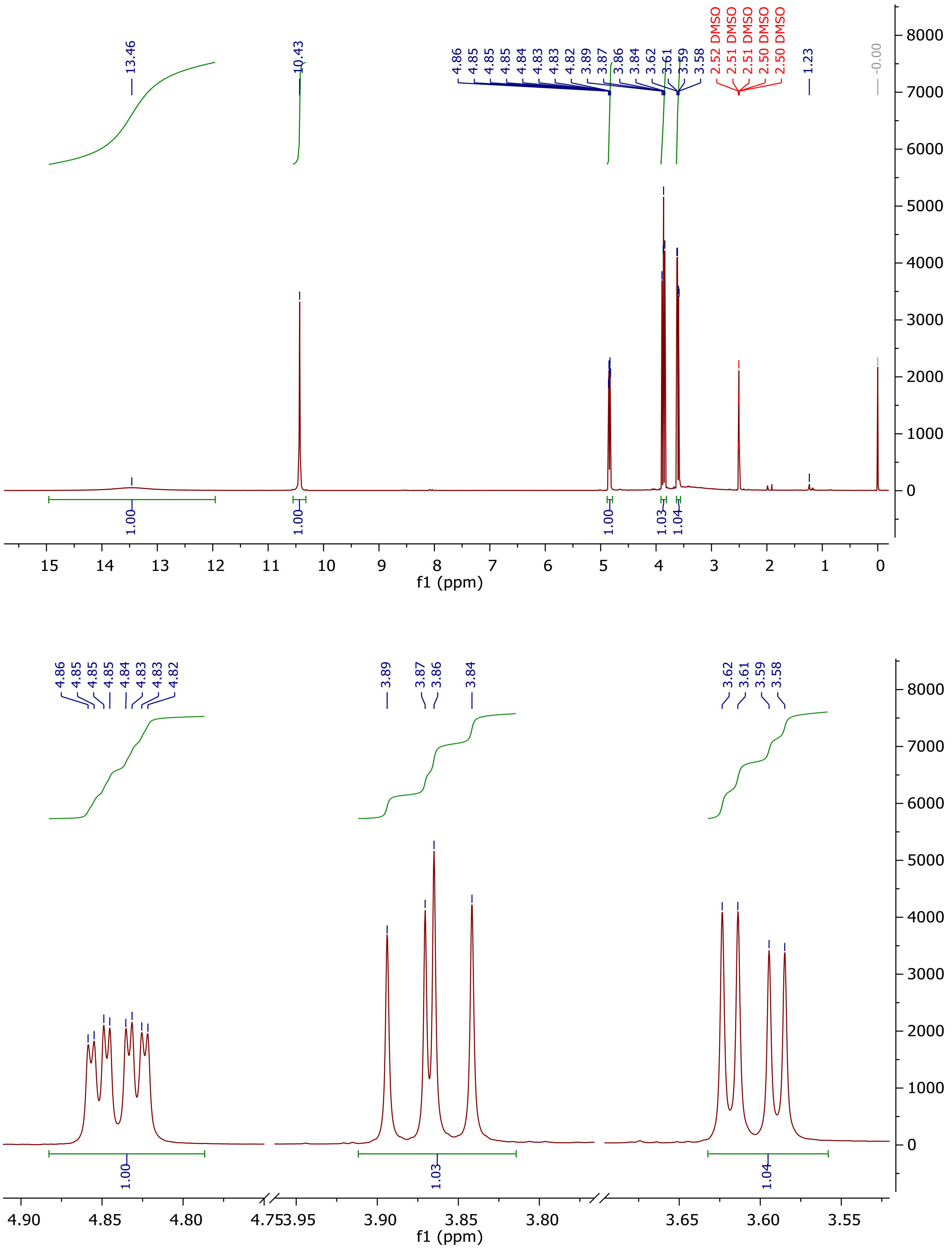

Fig. S16 ^{1}H NMR spectrum of 2-thioxothiazolidine-4-carboxylic acid (**3**) ($CDCl_3$, 400 MHz, 20 °C).

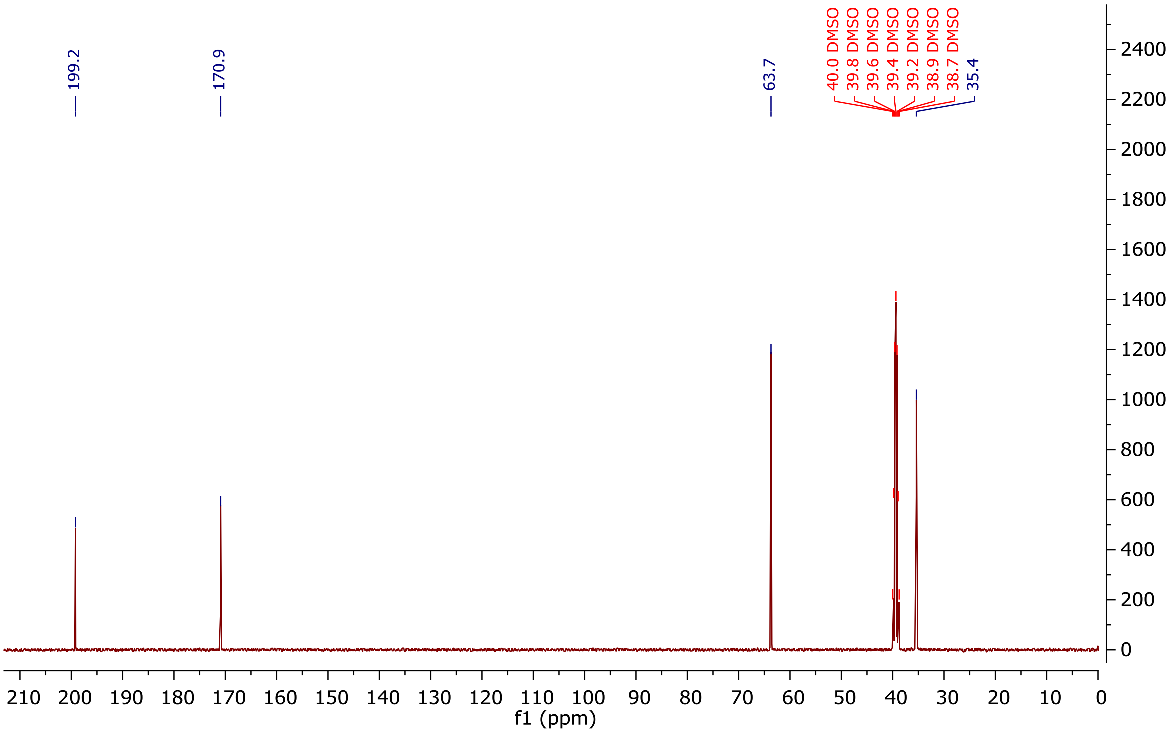

Fig. S17 ^{13}C NMR spectrum of 2-thioxothiazolidine-4-carboxylic acid (**3**) ($CDCl_3$, 100.6 MHz, 20 °C).

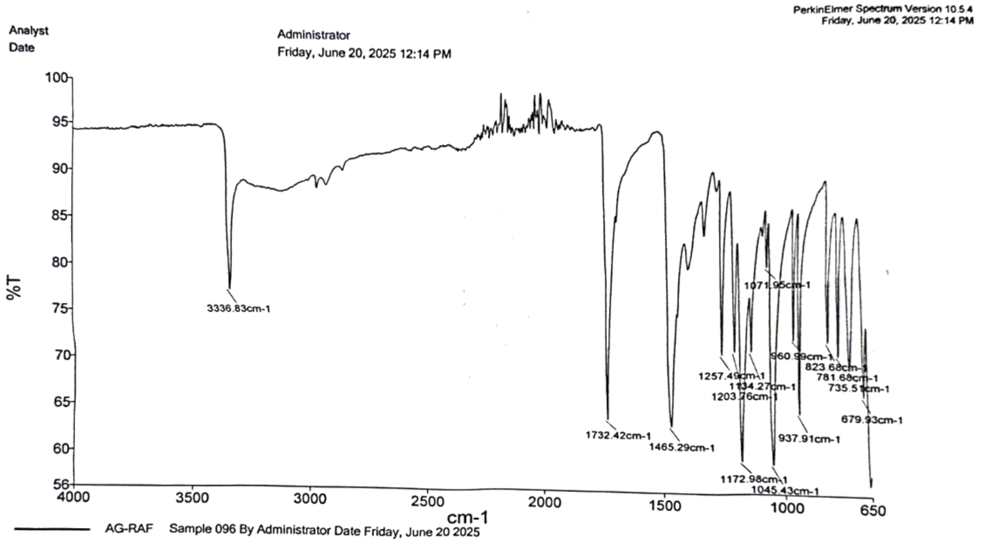

Fig. S18 IR spectrum of 2-thioxothiazolidine-4-carboxylic acid (**3**)

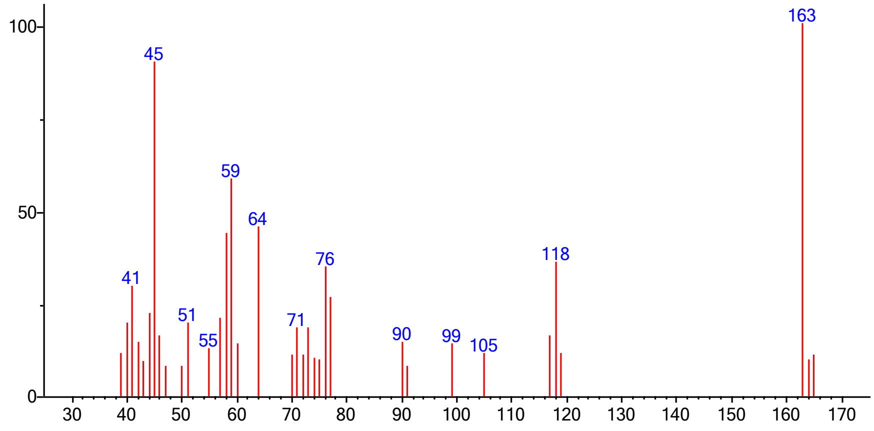

Fig. S19 EIMS spectrum of 2-thioxothiazolidine-4-carboxylic acid (**3**)

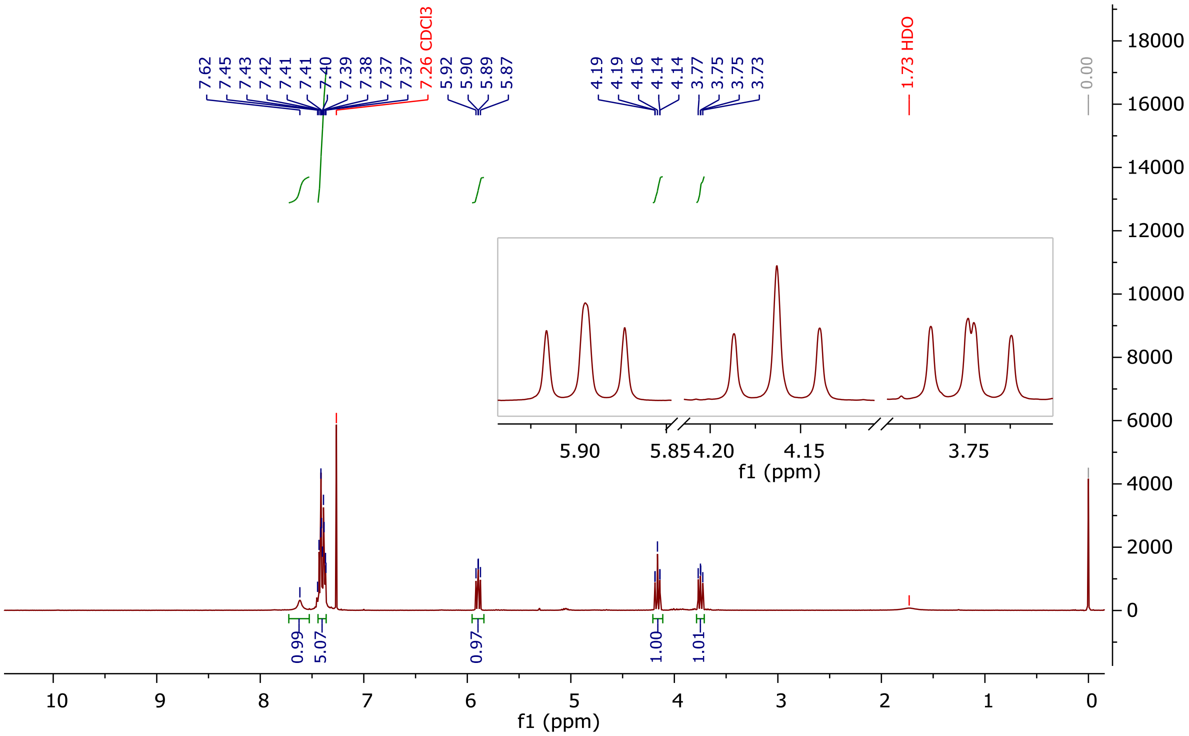

Fig. S20 ^{1}H NMR of *rac*-5-phenyl-1,3-oxazolidine-2-thione (*rac*-barbarin, **4**) ($CDCl_3$, 400 MHz, 20 °C)

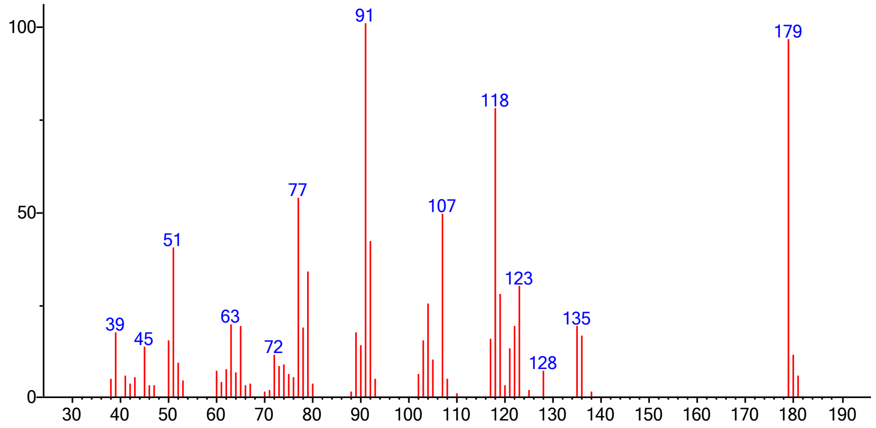

Fig. S21 EIMS spectrum of *rac*-barbarin (**4**)

Experimental procedures and characterization data and spectra of compounds 5–8

Experimental procedures, spectral and other analytical data of **5**, **6**, **7**, and **8** are provided here for completeness; the same data appear in the Supporting Information of a of a manuscript under review [14].

Isolation of (*S*)-5-phenyl-1,3-oxazolidine-2-thione ((*S*)-barbarin, 5)

(S)-5-Phenyl-1,3-oxazolidine-2-thione ((*S*)-barbarin, **5**) was isolated by preparative FDC on silica gel from the autolysate of stem and leaves, immediately after extraction from the plant material. The preparative separation of the autolysate (500 mg) was performed under isocratic conditions using 30% ethyl acetate in hexane (v/v) as the mobile phase, affording 85 mg of pure compound **5**. The purity of the isolated compounds was confirmed by TLC and GC/MS analysis. The spectral and other analytical data of the isolated barbarin were in agreement with previously published data [26,34]. Yellowish crystals. MP: 116–118 °C. RI (HP-5MS): 2069. FTIR (neat), cm^{-1}: 3181 (N–H), 3036 (C–H), 1734 (C=O), 1669, 1537, 1457, 1153, 757, 696. ^1H NMR ($CDCl_3$), δ: 3.74 (ddd, 1H, J = 10.0, 8.1, 0.8, H-4), 4.16 (ddd, 1H, J = 10.0, 9.1, 0.8, H-4), 5.88 (dd, 1H, J = 9.1, 8.1, H-5), 7.36 – 7.43 (m, 5H, Ar-H), 8.20 (brs, 1H, N–H). ^{13}C NMR ($CDCl_3$), δ: 51.2 (C-4), 84.0 (C-5), 126.0 (C-2', C-6'), 129.1 (C-3', C-5'), 129.4 (C-4'), 137.0 (C-1'), 189.5 (C-2). EIMS, m/z (rel. int.): 179 (95, $M^{+\bullet}$), 123 (30), 119 (27), 118 (77), 107 (49), 92 (42), 91 (100), 79 (33), 77 (53), 51 (40). For compound enumeration, see Figure 1.

Determination of absolute configuration of 5 via NMR titration using a chiral lanthanide shift reagent

The identification and differentiation of barbarin enantiomers were achieved through ^1H NMR titration with a chiral lanthanide shift reagent. This approach enabled the resolution of enantiomeric proton signals via formation of diastereomeric complexes.

Separation of ^1H NMR signals of barbarin enantiomers using a chiral lanthanide shift reagent

The identification and differentiation of barbarin enantiomers was achieved via NMR titration using a chiral lanthanide shift reagent. Synthetic (±)-barbarin, previously prepared in our laboratory [26], was titrated with europium(III) tris[3-(heptafluoropropylhydroxymethylene)-D-camphorate], $Eu(hfc)_3$ purchased from Sigma-Aldrich (St. Louis, MO, USA). A sample of (±)-barbarin (5 mg) was dissolved in 0.7 mL of C_6D_6, and standard ^1H NMR acquisition parameters were applied (32k time domain points, 10 ppm spectral width, O1 = 6.0 ppm, 45° pulse angle, 5 s acquisition time, and 16 scans). Following acquisition of the initial ^1H NMR spectrum, a known amount (2–4 mg) of $Eu(hfc)_3$ was added, and the spectrum was recorded again. Incremental additions of the shift reagent resulted in gradual resolution of the H-4a and H-4b proton signals, corresponding to the two enantiomers of barbarin, which formed diastereomeric complexes with $Eu(hfc)_3$ (see Figs. S20 and S21) The molar ratios of $Eu(hfc)_3$ to (±)-barbarin employed were 0.00, 0.07, 0.13, 0.20, and 0.41. Complete resolution of the C-4 proton signals for the two enantiomers was achieved at a $Eu(hfc)_3$-to-racemic barbarin molar ratio of 0.41. Enantiomeric composition (or enantiomeric excess) was calculated from the ratio of the signal integral of H-4a(S) or H-4b(S) to half the integral of the overlapping H-4a+b(R) signal, using the resolved ^1H NMR shifted spectra (see Figure 3, A5 in ref. [26]).

Subsequently, the same titration procedure was applied to determine the enantiomeric purity of barbarin isolated from the above- (stem and leaves) and parts' autolysate of *B. vulgaris*. In all plant-derived samples, only a single set of signals was observed, corresponding exclusively to one enantiomer, namely (*S*)-barbarin.

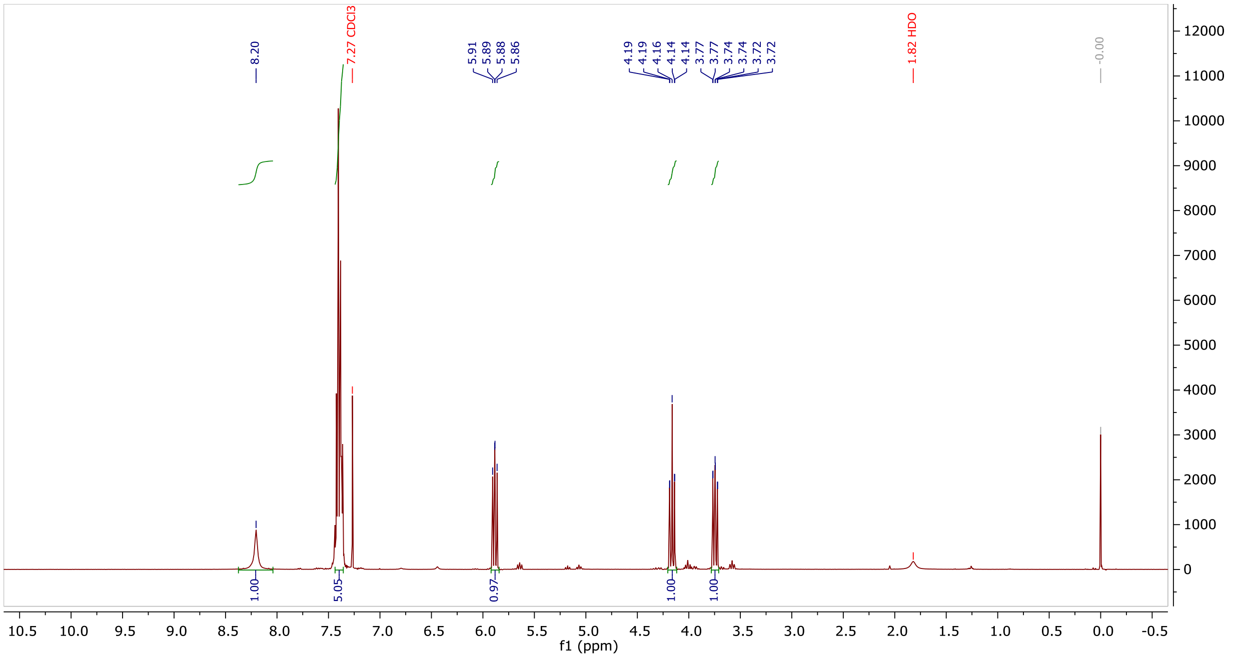

Fig. S22 ¹H NMR spectrum of (*S*)-5-phenyl-1,3-oxazolidine-2-thione (syn. barbarin) (**5**) ($CDCl_3$, 400 MHz, 20 °C).

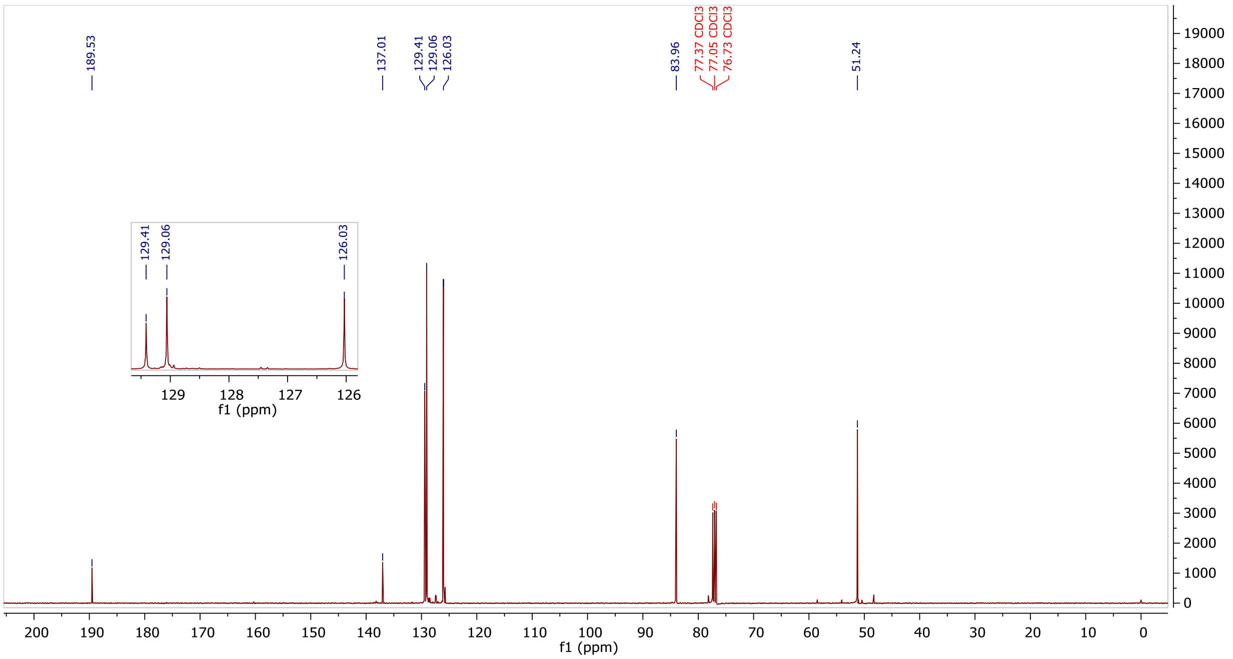

Fig. S23 ¹³C NMR spectrum of (*S*)-5-phenyl-1,3-oxazolidine-2-thione (syn. barbarin) (**5**) ($CDCl_3$, 100.6 MHz, 20 °C).

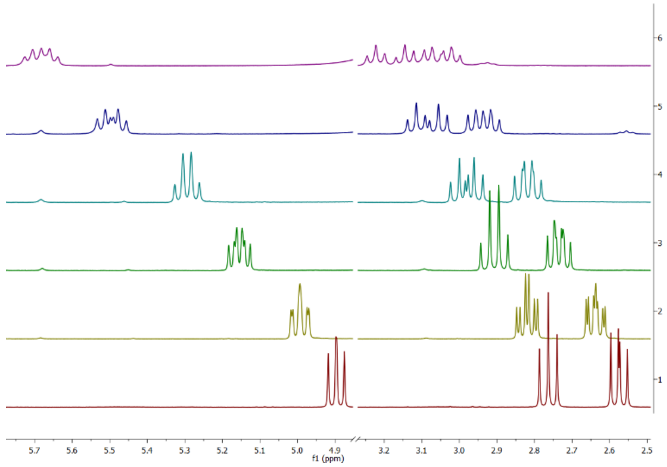

Fig. S24 Partial ^{1}H NMR spectra showing the chemical shift changes of the C-4a, C-4b, and C-5 proton signals upon incremental additions of Eu(hfc)$_3$ to a racemic mixture of barbarin, recorded in C_6D_6 at 400 MHz. The molar ratio of Eu(hfc)$_3$ to barbarin was: 0 (1), 0.069 (2), 0.13 (3), 0.21 (4), 0.31 (5), and 0.41 (6)

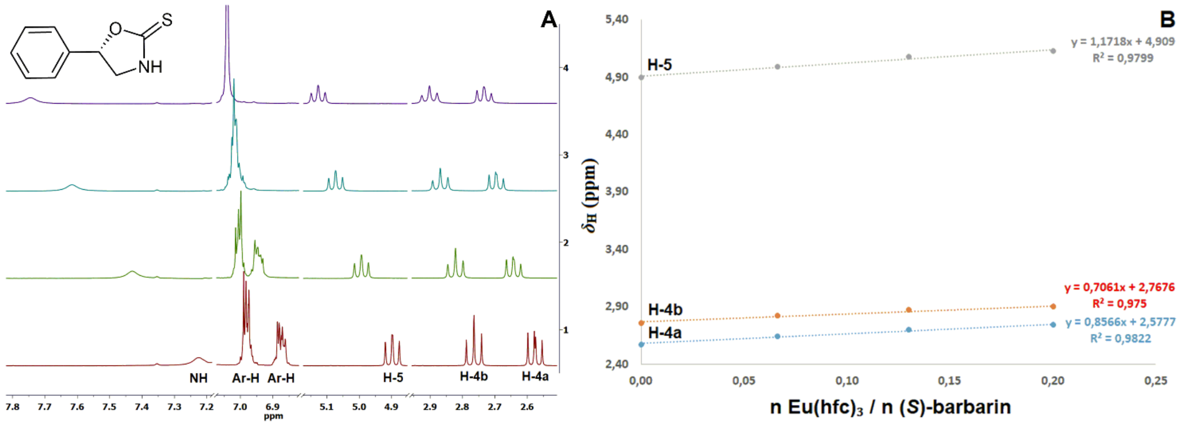

Fig. 25. A) The ^{1}H NMR signal shifts after incremental additions of Eu(hfc)$_3$ to isolated (*S*)-barbarin recorded in C_6D_6 (400 MHz). The molar Eu(hfc)$_3$/(*S*)-barbarin ratio was: 0 (1), 0.07 (2), 0.13 (3), and 0.20 (4). **B)** Relationship between δ_H values (ppm) and molar Eu(hfc)$_3$/(*S*)-barbarin ratio for H-4a, H-4b, and H-5 protons of (*S*)-barbarin with corresponding equations of the regression lines.

Isolation of 5-phenyl-1,3-thiazolidin-2-one (6)

Barbarea vulgaris W.T. Aiton (Brassicaceae) was collected at flowering in May 2015 near Ribariće (Tutin, Serbia). A voucher specimen (MD201514) was deposited at the Herbarium of the State University of Novi Pazar, and species identity was confirmed by a botanist. Aerial parts were washed, weighed, and immediately analyzed.

GLS hydrolysis was carried out using endogenous myrosinase. Fresh stems/leaves (250 g) were homogenized and mixed with water (500 mL). Et_2O (200 mL) was added and the mixture was incubated at 25 °C, 200 rpm, for 8 h. After adding NaCl (60 g), the organic layer was separated, filtered, and the residue re-extracted three times with DCM. Combined extracts were dried (Na_2SO_4) and the solvent was evaporated yielding 324 mg of the autolysate. Recrystallization of the autolysate from a 1:1 (v/v) mixture of Et_2O and hexane yielded a solid precipitate. The precipitate was filtered and washed with Et_2O, affording 80 mg of brownish crystals of compound **6**. The purity of the product was confirmed by TLC and GC/MS analysis. The spectral and other analytical data of the compound were consistent with previously published data [7,26,35,36].

Brownish crystals. Yield: 80 mg. RI (HP-5MS): 1912. FTIR (neat), cm^{-1}: 3298 (N–H), ≈1770 (C=O), 1645, 1492, 1191, 696. ^{1}H NMR ($CDCl_3$), δ: 3.31 – 3.41 (*m*, 1H, H-4), 3.49 – 3.56 (*m*, 1H, H-4), 4.51 (*"t"*, 1H, *J* = 7.8, H-5), 7.17 – 7.29 (*m*, 5H, Ar-H), 8,28 (*brs*, 1H, N–H). ^{13}C NMR ($CDCl_3$), δ: 45.7 (*t*, C-4), 47.3 (*d*, C-5), 127.7 (*d*, C-3', C-5'), 128.3 (*d*, C-2', C-6'), 128.8 (*d*, C-4'), 140.3 (*s*, C-1'), 165.0 (*s*, C-2). EIMS, m/z (rel. int.): 179 (73, $M^{+\bullet}$), 135 (21), 123 (100), 122 (72), 121 (72), 91 (51), 78 (21), 77 (28), 51 (26), 45 (24). For compound enumeration, see Figure 1.

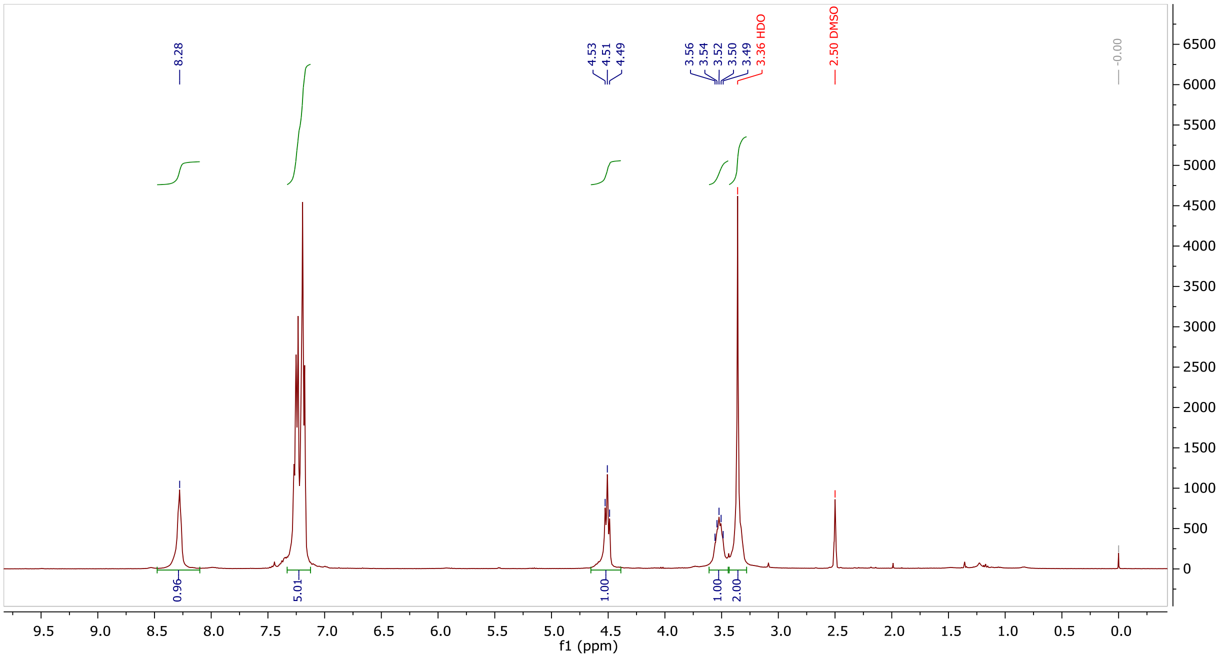

Fig. S26 ^{1}H NMR spectrum of 5-phenyl-1,3-thiazolidin-2-one (**6**) ($DMSO_{d6}$, 400 MHz, 20 °C).

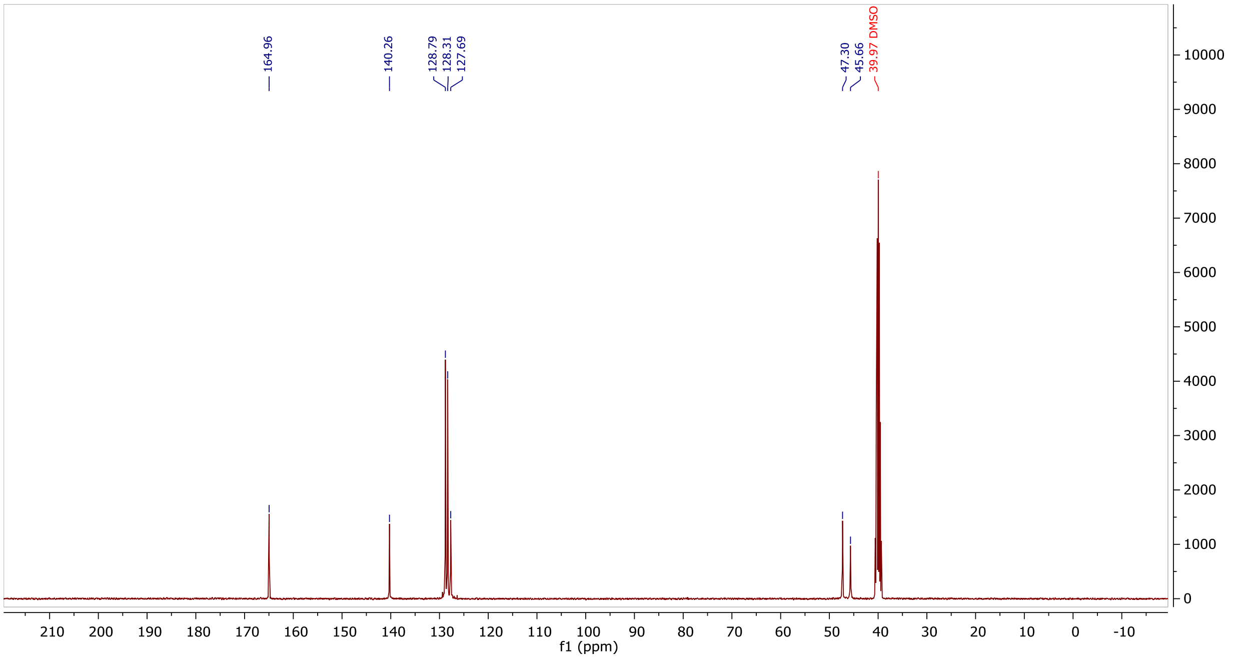

Fig. S27 ^{13}C NMR spectrum of 5-phenyl-1,3-thiazolidin-2-one (**6**) ($DMSO_{d6}$, 100.6 MHz, 20 °C).

Synthesis of (±)-5-phenyl-1,3-oxazolidin-2-one (resedine, 7)

5-Phenyl-1,3-oxazolidin-2-one (resedine, **7**) was synthesized starting from *rac*-2-amino-1-phenylethanol according to the procedure reported by Nicolas and co-workers [37].

A mixture of (±)-2-amino-1-phenylethanol (1 g, 7.3 mmol), diethyl carbonate (DEC, 1.76 mL, 14.5 mmol), and K_2CO_3 (0.152 g, 11 mmol) was heated at 130 °C with vigorous stirring in a distillation apparatus. Ethanol (1.5 mL) was distilled off, and the reaction mixture was allowed to cool to room temperature. The resulting white solid was dissolved in 50 mL of EtOAc, filtered, washed with brine (2 × 10 mL), dried over anhydrous sodium sulfate, and evaporated to dryness on a rotary evaporator at 40 °C, yielding 601 mg of crude product. Recrystallization of the crude product from a 1:1 (v/v) mixture of EtOAc and hexane afforded 208 mg of **7**. The purity of the synthesized compound was confirmed by TLC and GC/MS analysis. The spectral data of the synthesized product were in agreement with previously published data [26,35,38].

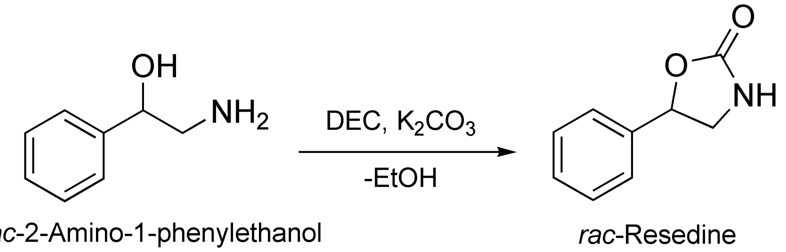

Fig. S28 Synthetic scheme leading to *rac*-5-phenyl-1,3-oxazolidin-2-one (**7**)

White powder. Yield: 208 mg (18%). MP: 88.5 – 89.0 °C. RI (HP-5MS): 1785. FTIR (neat), cm^{-1}: 3264 (N–H), 1715 (C=O), 1239, 694. ^1H NMR ($CDCl_3$), δ: 3.55 (*dd*, 1H, *J* = 8.7, 7.7, H-4), 3.98 (*t*, 1H, *J* = 8.7, H-4), 5.62 (*dd*, 1H, *J* = 8.7, 7.7, H-5), 6.39 (*brs*, 1H, NH), 7.34 – 7.44 (*m*, 5H, Ar-H). ^{13}C NMR ($CDCl_3$), δ: 48.4 (*t*, C-4), 77.9 (*d*, C-5), 125.7 (*d*, C-2', C-6'), 128.9 (*d*, C-3', C-4', C-5'), 138.4 (*s*, C-1'), 160.1 (*s*, C-2). EIMS, m/z (rel. int.): 163 (17, $M^{+\bullet}$), 118 (11), 107 (100), 105 (13), 91 (17), 89 (9), 79 (42), 78 (9), 77 (22), 51 (15). For compound enumeration, see Figure 1.

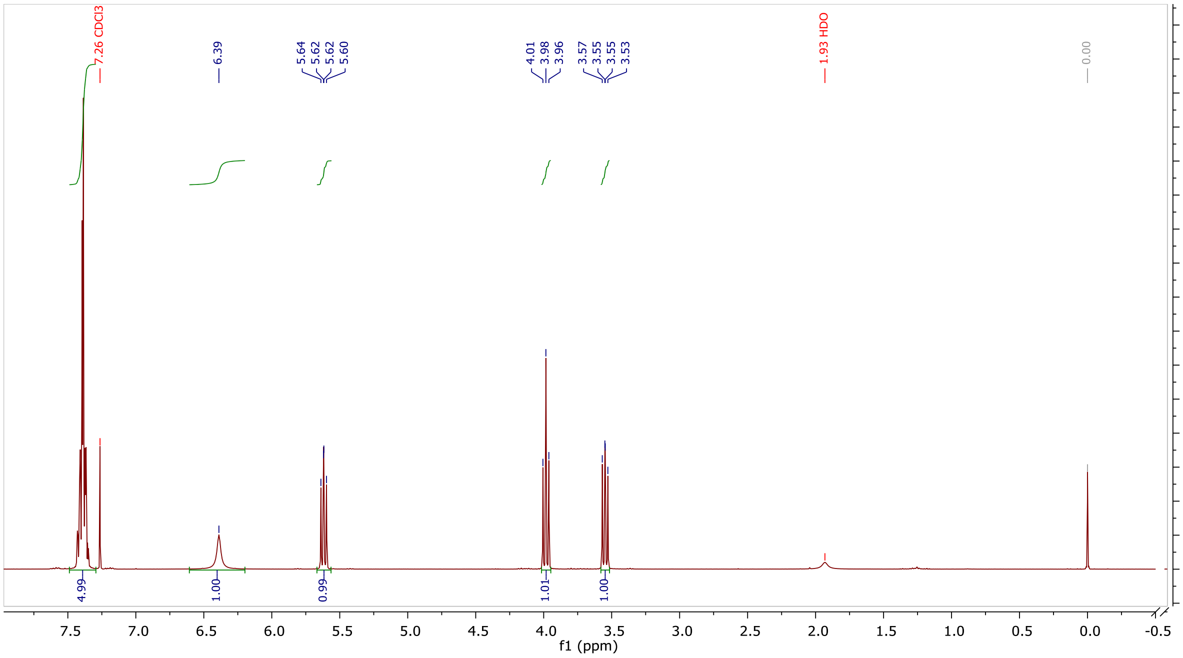

Fig. S29 ^{1}H NMR spectrum of 5-phenyl-1,3-oxazolidin-2-one (resedine) (**7**) ($CDCl_3$, 400 MHz, 20 °C).

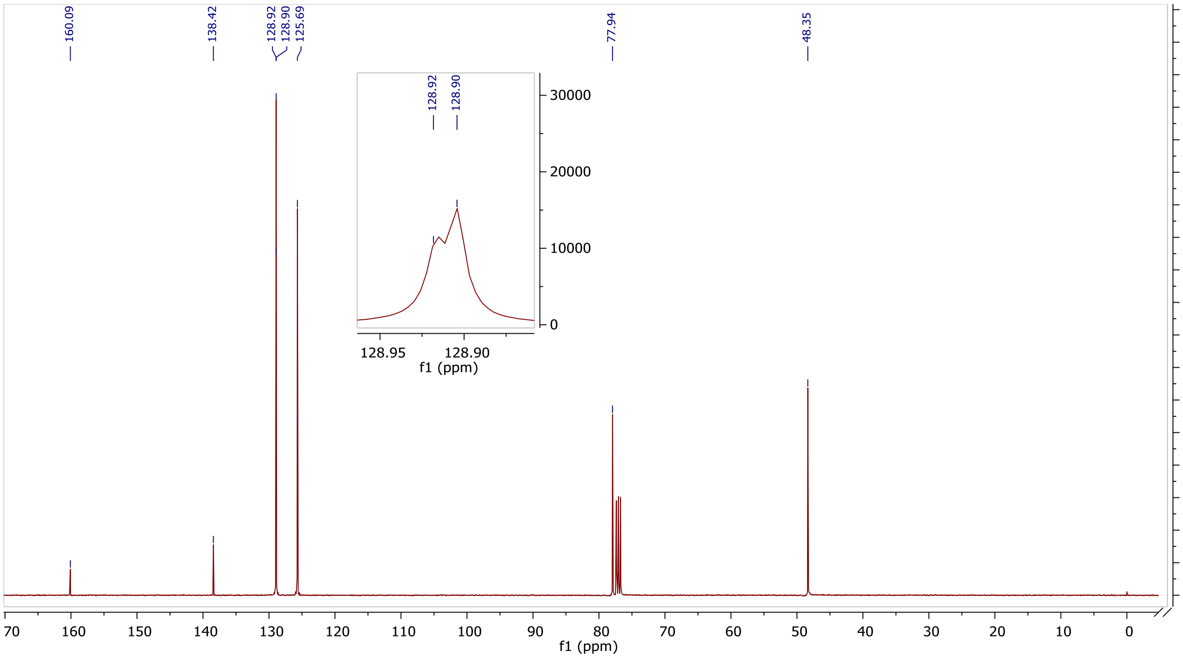

Fig. S30 ^{13}C NMR spectrum of 5-phenyl-1,3-oxazolidin-2-one (resedine) (**7**) ($CDCl_3$, 100.6 MHz, 20 °C).

Synthesis of 2-(4-hydroxyphenyl)ethyl isothiocyanate (8)

2-(4-Hydroxyphenyl)ethyl isothiocyanate (**8**) was synthesized from *p*-tyramine following the procedure described by Spencer et al. [39]. Namely, to a solution of commercially available *p*-tyramine (1 eq, 600 mg) in 30 mL of anhydrous THF, Et_3N (2 eq, 1.23 mL) and CS_2 (5 eq, 1.32 mL) were added. The mixture was stirred at 30–40 °C for 1 hour. After cooling to 0–10 °C, hydrogen peroxide (30% aq, 2.8 eq, 1.25 mL) was added dropwise. Concentrated HCl was then added to adjust the pH to 1.0–1.5. The resulting precipitated sulfur was filtered off, and the filtrate was extracted with EtOAc (3 × 50 mL), dried over anhydrous sodium sulfate, and concentrated to yield a yellow oil. The crude product was purified by flash column chromatography on silica gel (30% Et_2O/hexane, isocratic conditions), affording 395 mg of the pure **8** in 50% yield. The purity of the synthesized isothiocyanate **8** was confirmed by TLC and GC/MS analysis. The spectral data of the synthesized compound matched previously published data [39,40].

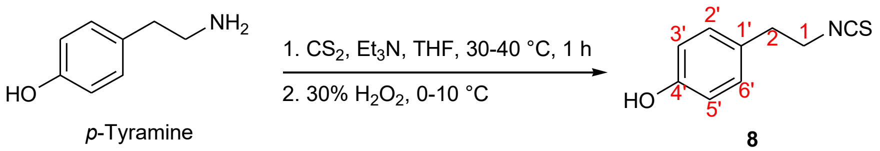

Fig. S31 Synthetic scheme leading to the 2-(4-hydroxyphenyl)ethyl isothiocyanate (**8**)

*2-(4-Hydroxyphenyl)ethyl isothiocyanate (**8**).* Light yellow oil. RI (HP-5MS): 1799. FTIR (neat), cm^{-1}: 3396 (O–H), 2184, 2090 (N=C=S), 1613, 1514, 1440, 1223 (arC–OH). ^1H NMR ($CDCl_3$), δ: 2.91 (*t*, 2H, J = 6.9, H-2), 3.67 (*t*, 2H, J = 6.9, H-1), 4.40 (*brs*, 1H, OH), 6.79 – 6.82 (*m*, 2H, H-3', H-5'), 7.06 – 7.10 (*m*, 2H, H-2', H-6'). ^{13}C NMR ($CDCl_3$), δ: 35.6 (*t*, C-2), 46.6 (*t*, C-1), 115.6 (*d*, C-3', C-5'), 129.2 (*s*, C-1'), 130.0 (*d*, C-2', C-6'), 130.5 (NCS), 154.6 (*s*, C-4'). EIMS, *m*/*z* (rel. int.): 181 (1.3), 180 (1.8), 179 (15, $M^{+\bullet}$), 109 (2), 108 (12), 107 (100), 77 (2), 55 (2), 51 (2), 39 (2).

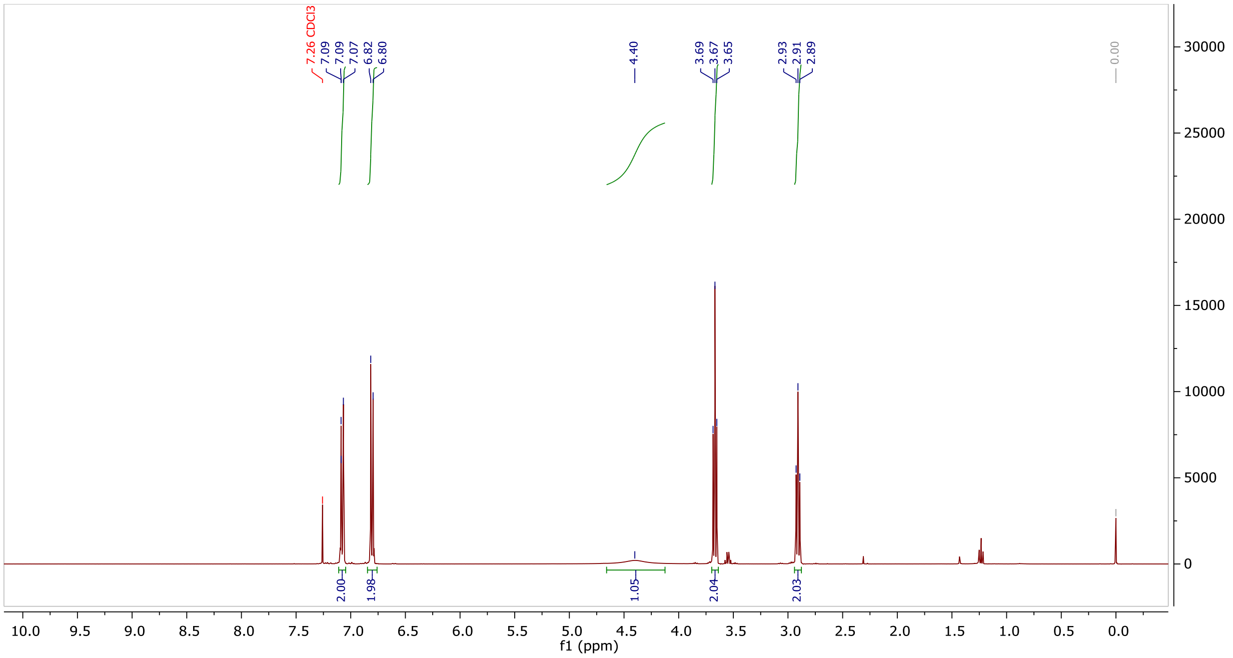

Fig. S32 ^{1}H NMR spectrum of 2-(4-hydroxyphenyl)ethyl isothiocyanate (**8**) ($CDCl_3$, 400 MHz, 20 °C).

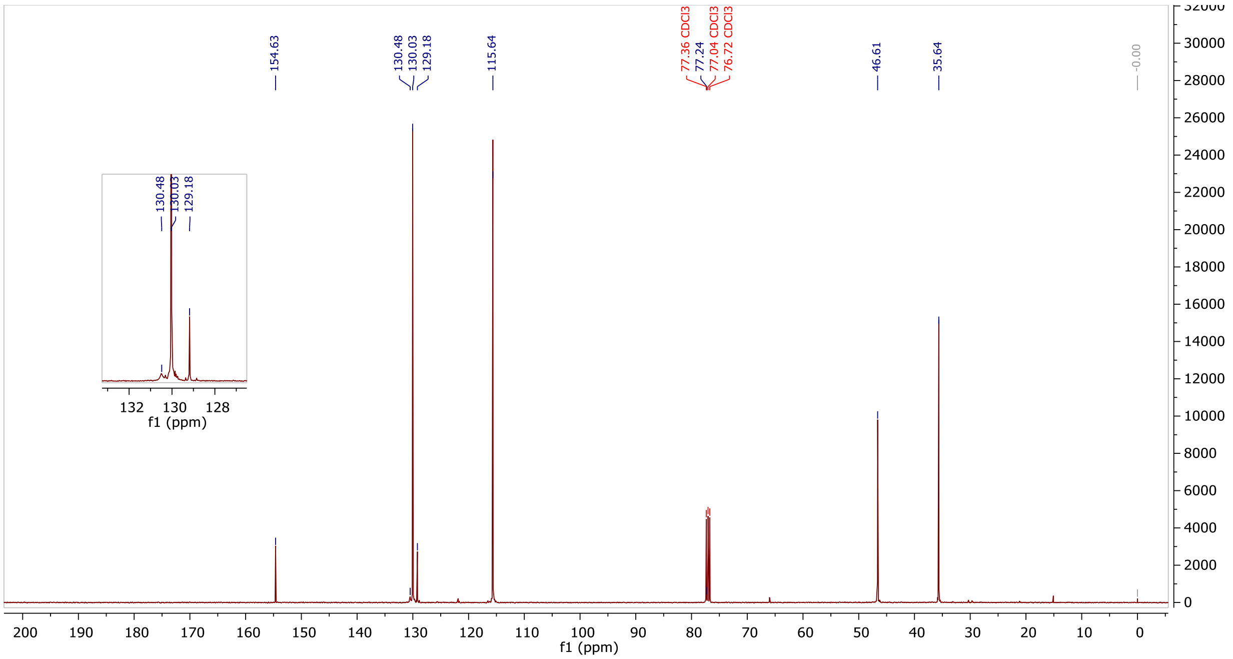

Fig. S33 ^{13}C NMR spectrum of 2-(4-hydroxyphenyl)ethyl isothiocyanate (**8**) ($CDCl_3$, 100.6 MHz, 20 °C).
